# Single-dose GC101 gene therapy for spinal muscular atrophy types II and III: an open-label single-arm study

**DOI:** 10.1007/s12519-025-00955-x

**Published:** 2025-08-04

**Authors:** Xiu-Wei Ma, Xin-Yang Jiang, Zhong-Qiu Li, Xiao-Yan Dong, Wen-Hao Ma, Yong-Xia Wang, Shuang-Qing Yu, Rui-Jie Gu, Xiao-Dong Wang, Bai-Mao Zhong, Fang He, Juan Xu, Ying-Ying Mao, Yan-Ping Zhang, Shan Zhang, Ting Li, Chong-Yang Li, Sheng Zhang, Xiao Yang, Li-Na Zhu, Zhi-Jie Wu, Bing Zhou, Lu Zhuang, Qiu-Ping Li, Xiao-Bing Wu, Zhi-Chun Feng

**Affiliations:** 1 Department of Pediatrics, Faculty of Pediatrics, The Seventh Medical Center of the Chinese PLA General Hospital, Beijing 100700, China; 2 National Engineering Laboratory for Birth Defects Prevention and Control of Key Technology, Beijing 100700, China; 3 Beijing Key Laboratory of Pediatric Organ Failure, Beijing 100700, China; 4 Genecradle Therapeutics Inc., Beijing 100176, China; 5 Department of Pediatrics, Dongguan Children’s Hospital, Guangdong 523325, China; 6 Department of Pharmacy, Medical Supplies Center of the Chinese PLA General Hospital, Beijing 100700, China; 7 Advanced Innovation Center for Big Data-Based Precision Medicine, Beihang University, Beijing 100191, China; 8 State Key Laboratory of Kidney Diseases, Beijing 100853, China

## Abstract

**Supplementary Information:**

The online version contains supplementary material available at 10.1007/s12519-025-00955-x.

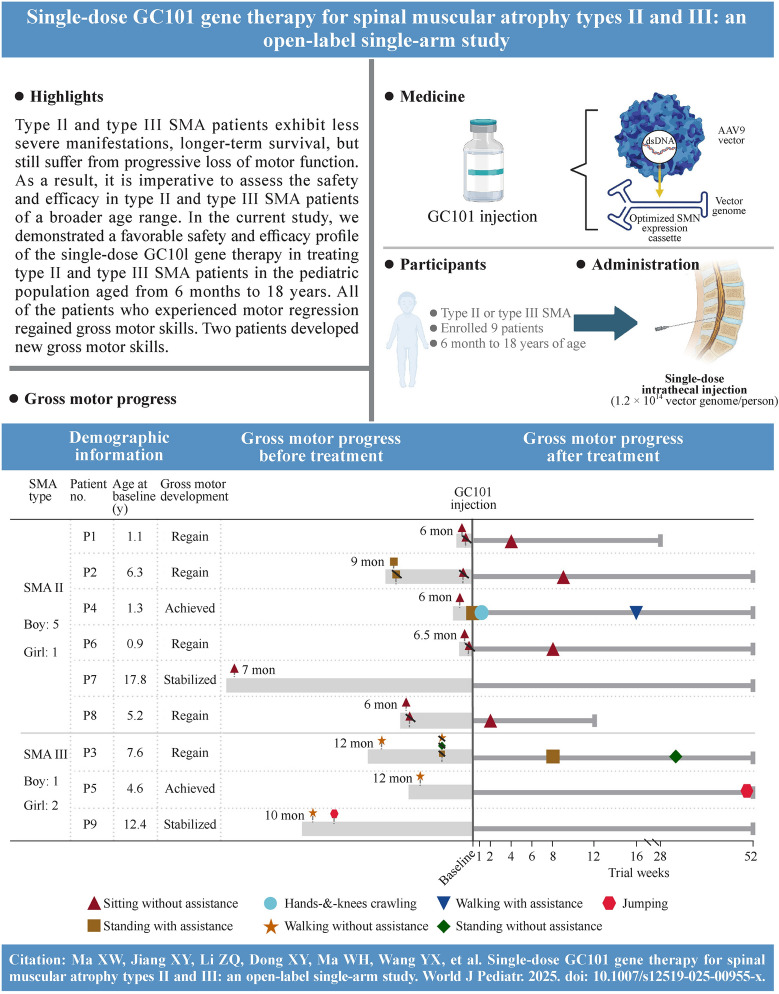

Spinal muscular atrophy (SMA) is a progressive autosomal recessive motor neuron disease caused by a deficiency of survival motor neuron (SMN) protein due to biallelic deletion or a pathogenic variant of the *SMN1* gene [1], which is classified into five types on the basis of onset age and severity, and the severity generally correlates inversely with the copy number of *SMN2*, a homologous gene on chromosome 5q13 [2]. Type II SMA patients acquire independent sitting but are unable to walk or stand independently [3]. Patients with type III SMA achieve independent walking and are affected mainly by a tendency to fall, lower-limb weakness and fatigue [4]. If not treated, patients with types II and III SMA experience overall deterioration in motor function as the disease progresses [5]. Currently, only three therapies have been approved for the treatment of SMA patients, including nusinersen, onasemnogene abeparvovec, and risdiplam [1].

Our team developed GC101, a recombinant adeno-associated viral serotype 9 (AAV9) gene therapy that has been used in type I SMA patients [6] and aims to correct SMN deficiency by delivering the normal target gene into target organs to express the functional protein. Onasemnogene abeparvovec, a drug in the same class reported effectiveness and tolerance in sitting, nonambulatory SMA patients < 60 months of age [7]. However, as types II and III SMA patients present less severe manifestations and longer-term survival [1], it is imperative to assess the safety and efficacy in types II and III SMA patients across a broader age range.

Here, we conducted a single-arm, open-label clinical study (ChiCTR2100054441) to evaluate the safety and efficacy of GC101 therapy via a single-dose intrathecal (IT) injection for types II and III SMA patients aged 6 months to 18 years over a 52-week follow-up. Patients were scheduled to receive GC101 at the Seventh Medical Center of Chinese PLA General Hospital. Written informed consent was obtained from the patients’ parents or guardians before enrollment. GC101 was administered as a single-dose IT injection of 1.2 × 10^14^ vector genomes/person by professional physicians. The details of the study design, ethics*,* inclusion and exclusion criteria, treatments, and outcomes are listed in the protocol in the Supplementary materials.

Nine patients (P1-P9) received GC101 administration from April 18, 2022, to January 19, 2023 (Fig. 1a). The demographic and clinical characteristics of the patients at baseline are shown in Table 1. Six patients (P1, P2, P4, P6, P7, and P8) were diagnosed with type II SMA, and three patients (P3, P5, and P9) were diagnosed with type III SMA. No patient required tube feeding or ventilatory support during the follow-up period.

**Fig. 1:**
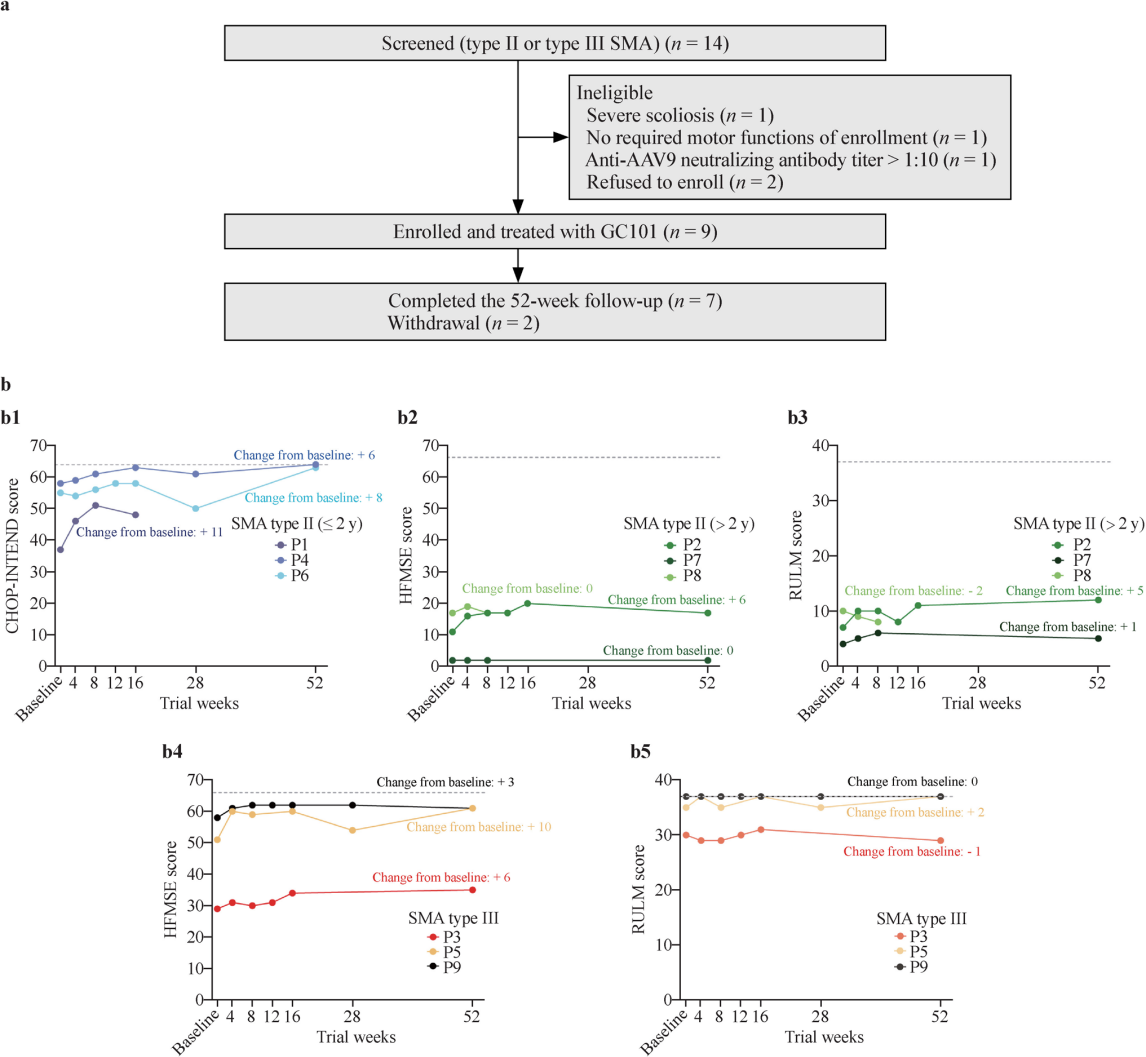
Flow diagram and efficacy of GC101. **a** Flow chart of the study; **b** changes in CHOP-INTEND, HFMSE, and RULM scores in nine patients. Overall changes in the CHOP-INTEND, HFMSE, and RULM scores of the nine patients are shown in the line chart. The score changes between the baseline value and the value at the end of follow-up are presented in words beside the line, with different colors used for patient differentiation. **b1** Changes in the CHOP-INTEND score in type II SMA patients younger than two years of age. The gray horizontal dashed line indicates the highest CHOP-INTEND score of 64; **b2** change in the HSFME score in type II SMA patients older than two years of age. The gray horizontal dashed line indicates the highest HFMSE score of 66; **b3** change in RULM score in type II SMA patients older than two years of age. The gray horizontal dashed line indicates the highest RULM score of 37; **b4** change in the HSFME score in type III SMA patients. The gray horizontal dashed line indicates the highest HSFME score of 66; **b5** change in RULM score in type III SMA patients. The gray horizontal dashed line indicates the highest RULM score of 37. *CHOP-INTEND* Children’s Hospital of Philadelphia Infant Test of Neuromuscular Disorders, *HFMSE* Hammersmith Functional Motor Scale-Expanded, *RULM* Revised Upper Limb Module, *SMA* spinal muscular atrophy

**Table 1:** Baseline demographic and clinical characteristics of nine patients

Variables	Type II SMA patients (*n* = 6)						Type III SMA patients (*n* = 3)		
	≤ 2 y (*n* = 3)			> 2 y (*n* = 3)					
	P1	P4	P6	P2	P7	P8	P3	P5	P9
Demographic characteristics									
Age of onset (y)	0.75	0.50	0.42	0.75	1.00	0.50	1.17	2.00	1.25
Age at baseline (y)	1.06	1.30	0.94	6.29	17.79	5.16	7.57	4.61	12.38
Motor function assessment									
CHOP-INTEND (score)	37	58	55	–	–	–	–	–	–
HFMSE (score)	–	–	–	11	2	17	29	51	58
RULM (score)	–	–	–	7	4	10	30	35	37
6MWT (meters)	–	–	–	–	–	–	–	138	495
Ambulatory status	N	N	N	N	N	N	N	Y	Y
Gross motor skills at baseline									
Sitting without assistance	N	Y	N	N	Y	N	Y	Y	Y
Standing with assistance	N	N	N	N	N	N	N	Y	Y
Walking without assistance	N	N	N	N	N	N	N	Y	Y
Motor regression	Y	N	Y	Y	N	Y	Y	N	N
SMN2 copy number	2	2	3	3	3	3	3	3	3
Clinical characteristics									
Ventilatory support	N	N	N	N	N	N	N	N	N
Nonoral feeding support	N	N	N	N	N	N	N	N	N

*SMA* spinal muscular atrophy, *HFMSE* Hammersmith Functional Motor Scale-Expanded, *RULM* revised upper limb module, *CHOP-INTEND* Children's Hospital of Philadelphia Infant Test of Neuromuscular Disorders, *6MWT* six-minute walk test, *Y* yes, *N* no

The primary objective of this study was to assess safety, including adverse events (AEs) and laboratory tests. AEs were evaluated throughout the 52-week follow-up period (Supplementary Table 1). No deaths or patient withdrawals due to AEs were reported. A serious adverse event (SAE) was reported in P3 due to pneumonia at week 28 after receiving GC101, which was considered unrelated to the use of GC101. Three patients (P1, P3, and P6) experienced five grade III AEs, all of which were related to the respiratory system. One patient (P5) had lumbar puncture-associated complications [8] that were mild or moderate in severity and temporally associated with GC101 injection. High total cholesterol, triglyceride (TG), and low-density lipoprotein cholesterol (LDL-C) were observed in eight patients (Supplementary Fig. 1), and most of these events were temporally associated with the use of prednisolone during the first eight weeks after GC101 injection, which has been reported to influence fat metabolism [9]. An increase in aspartate aminotransferase (AST) was observed at P7 at week 6 (Supplementary Fig. 2). No specific medical interventions were required since the increase in AST was asymptomatic, and all the patients maintained LDL-C < 6.46 mmol/L with TG < 5.65 mmol/L. No AEs related to platelet level (Supplementary Fig. 3) or cardiac troponin I (Supplementary Fig. 4) were observed in the current study. The vector DNA level in the blood increased sharply after GC101 injection, remained stable for 1–8 weeks, and then gradually decreased until it reached less than the lower limit of quantification at week 52 (Supplementary Fig. 5).

The secondary objective was to evaluate the efficacy of GC101. Clinically significant responses were defined as at least having a four-point increase from the baseline value of the Children’s Hospital of Philadelphia Infant Test of Neuromuscular Disorders (CHOP-INTEND), a three-point increase in Hammersmith Functional Motor Scale-Expanded (HFMSE), a two-point increase in the Revised Upper Limb Module (RULM), and a 30-m improvement in the 6-minute walk test (6MWT) [10]. CHOP-INTEND was used to assess motor function in type II SMA patients younger than two years of age (P1, P4, and P6). P1 had an 11-point increase from the baseline value at week 16 before withdrawal. P4 had a 6-point increase, and P6 had an 8-point increase from the baseline at week 52 (Fig. 1b1).

HFMSE (Fig. 1b2 and b4) and RULM (Fig. 1b3 and b5) were used to assess motor function in types II and III SMA patients older than two years of age (type II: P2, P7, P8; type III: P3, P5, P9). Four patients showed clinically meaningful improvement in their HFMSE scores (P2 and P3: 6-point increase, P5: 10-point increase, P9: 3-point increase). P2 and P5 each showed clinically meaningful improvements in the RULM score (5-point increase). P7 (aged 17.79 years at baseline) showed no change in HFMSE and a 1-point increase from the baseline value of RULM during the follow-up period**.** The P9 score stabilized at the highest score until the end of follow-up. A 1-point decrease was observed in P3, which was within the stable range (− 2 to + 2) [11].

The two ambulatory patients were further evaluated by the 6MWT, and both showed clinically meaningful improvement. At baseline, P5 walked 138 m, and the distance increased to 290 m at week 52 (a 152-m increase), whereas P9 walked 495 m and reached 542 m at week 52 (a 47-m increase). P5 was found to have a waddling gait at 3.5 years of age, and P9 was found at 9 years of age. During the observation period, both P5 and P9 improved in waddling gait through GC101 treatment (Supplementary Videos 7 and 8).

All five patients (P1, P2, P3, P6, and P8) who experienced motor regression regained gross motor skills. After being treated with GC101, P3 regained the gross motor skills of standing with support at week 8 and standing without support at week 32. P4 achieved new gross motor milestones of crawling on hands and knees at week 1 and walking with assistance at week 16 after GC101 injection. P5 achieved the gross motor milestone of jumping at the end of the 52-week follow-up, and P9 improved from jumping forward to jumping up onto the plyometric jump box at week 23 after GC101 injection (Fig. 2 and Supplementary videos).

**Fig. 2:**
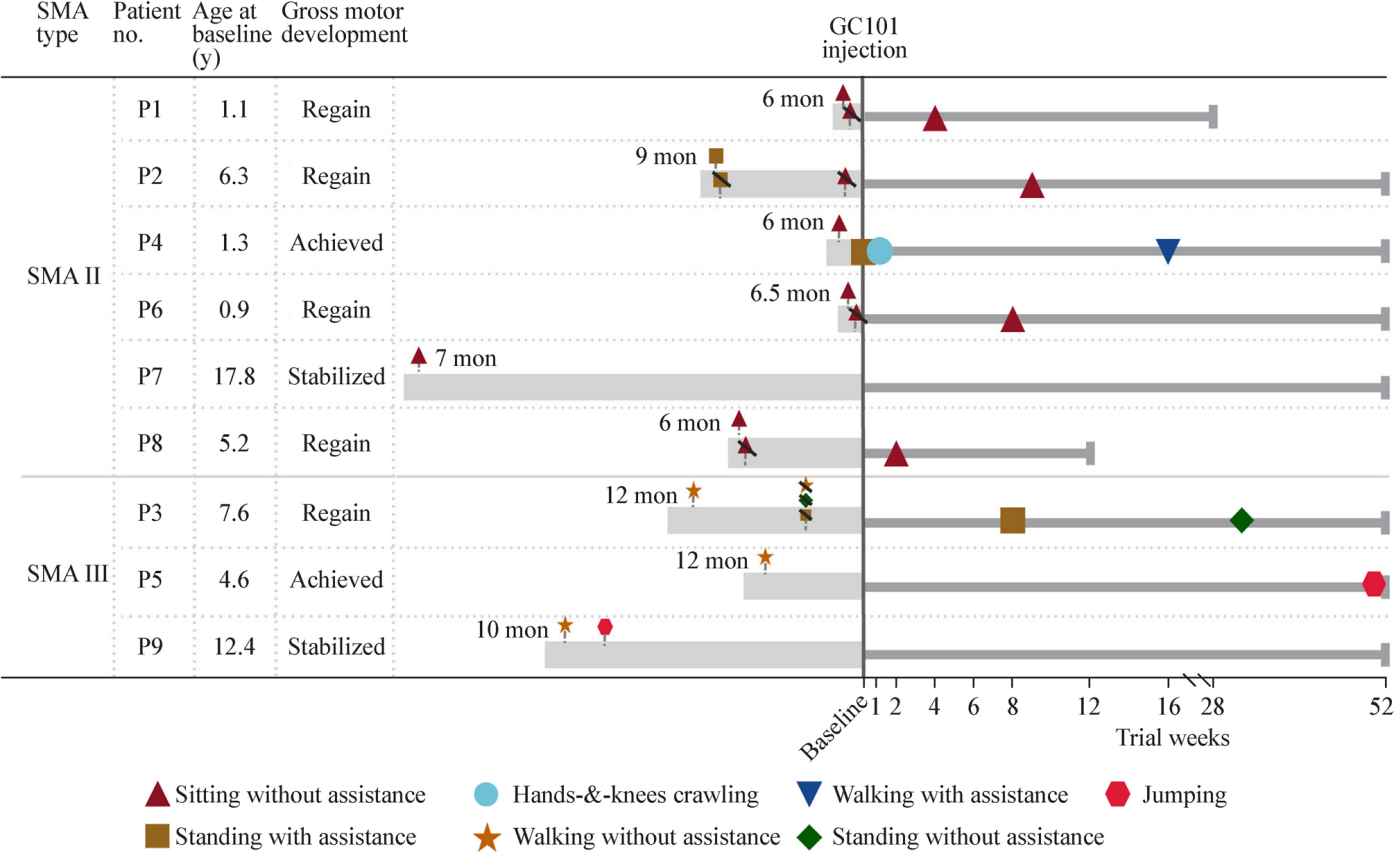
Gross motor progress in nine patients. The ages of nine patients with type II or type III spinal muscular atrophy (SMA) before GC101 injection and the status of gross motor progress throughout the follow-up period are shown in table format. The length of the gray bars on the left side indicates the age of the patients. The time at which motor milestones are achieved before enrollment is shown by the dashed line at the bottom, and the patient age at that time point is given next to the dashed line. The loss of motor skills is shown by adding a slash on the corresponding symbol. The regain of gross motor skills and the achievement of gross motor milestones after GC101 injection are shown by symbols on the following timeline. The bold timeline also indicates the follow-up status: P1 withdrew at week 28, P8 withdrew at week 12, and all other patients completed the 52-week follow-up. Different shapes indicate different motor functions (orange star: walking without assistance; red triangle: sitting without assistance; brown square: standing with assistance; blue circle: hands and knees crawling; dark blue inverted triangle: walking with assistance; dark green diamond: standing without assistance; pink hexagon: jumping)

The current study demonstrated that single-dose IT injection of GC101 was safe and effective for treating patients aged 6 months to 18 years who were diagnosed with type II or type III SMA. Motor function either improved or remained stable in all nine patients after GC101 treatment. No drug-related SAEs were observed in patients with SMA type II or type III. Similarly, our previous clinical trial in type I SMA patients reported no drug-related SAEs [6], demonstrating the favorable safety profile of GC101.

All possible drug-related AEs were transient and mild or moderate in severity. Four of the five respiratory grade III AEs occurred in two type II SMA patients (P1 and P6), which is consistent with the known respiratory issues in type II SMA patients [12]. AST elevation is frequently reported in AAV-mediated therapy. In our trial, the AE related to the increase in AST was reported only once during the 52-week observation period. None of the following AEs that were reported in a previous clinical trial that adopted AAV-mediated gene therapy, including hepatotoxicity, thrombocytopenia, microvascular thrombosis, and dorsal root ganglion [13], were observed in our patients.

Among all seven patients who completed the 52-week observation, six benefited with respect to motor function, which demonstrated the effectiveness of GC101 by comparing with a mean HFMSE decrease of 1.22 in a 12-month natural history study of types II and III SMA patients aged 30 months to 30 years [14]. Two patients (P2 and P5) presented clinically significant increases in the RULM score, and the upper limb functions of the other patients were still within the stable range [11]. The natural history data indicate that for types II and III SMA patients aged 30 months to 49 years, RULM exhibited a mean decrease of 0.41 over a 12-month period [11], which also proves the effectiveness of GC101.

Untreated older types II and III SMA patients present with motor function regression over time [15], and little evidence of regaining gross motor skills has been reported in previous clinical and real-world studies. Encouragingly, all patients who experienced motor function regression regained gross motor skills in our series. In addition to gross motor milestones, the results of the 6MWT showed clinically significant improvements from baseline in ambulatory patients, which demonstrated the effectiveness of GC101 by comparing the improvements with the mean decrease of 1.5 m in a 12-month natural history of type III SMA patients aged 0–50 years [16].

Specifically, P7 was the oldest (17.79 years) of all patients in our study whose HFMSE score remained unchanged throughout the entire observation period. The improvement in motor function shown by the changes in the HFMSE and RULM scores demonstrated clinical significance but was heterogeneous. Compared with earlier SMN protein supplementation in SMA model mice, later supplementation resulted in a greater number of partially innervated endplates and fewer fully innervated endplates [17]. Together with evidence from clinical trials that treatment prior to symptom onset results in improved developmental outcomes and greater functional independence [13, 15], these findings suggest that presymptomatic treatment may provide greater benefits to patients. We also found that the older the treatment age was, the smaller the increase in the HFMSE score from baseline to the end of the observation period (Supplementary Fig. 6), which is similar to the findings of other studies [18–20], indicating that the age at which treatment is initiated is a critical factor affecting the therapeutic response and outcome.

Arranging the vaccination schedule for children receiving gene therapy during the follow-up period is an issue worth exploring. To date, no dedicated studies have systematically investigated the optimal interval between vaccination and the administration of gene therapy. On the basis of three key pharmacological factors, (1) the inherent immunogenicity of AAV vectors has the potential to induce host immune responses following GC101 administration; (2) our study included an 8-week schedule of prednisolone usage; and (3) current guidelines recommend a 2- to 4-week interval between glucocorticoid (prednisolone) discontinuation and vaccination [21], and we recommend a conservative vaccination interval of 3 months after treatment.

This study has certain limitations. First, the sample size was relatively small, and the patient cohort exhibited heterogeneity in age and disease severity, which limits the generalizability of the findings to specific subgroups. Additionally, the observation period was relatively short, and this study did not include clinical or electrophysiological assessments of dorsal root ganglion pathology. Owing to the COVID-19 pandemic, a small number of patients missed hospital visits, leading to interruptions in follow-up observations.

In conclusion, our study demonstrated that single-dose IT injection of AAV9-mediated GC101 is safe and effective for treating patients with types II and III SMA. Further studies with larger sample sizes and longer observation periods deserve to be carried out to further understand the safety and efficacy of GC101 in types II and III SMA patients.

## Supplementary Information

Below is the link to the electronic supplementary material.

Supplementary file 1 (PDF 6682 KB)

Supplementary file 2 (MP4 13215 KB)

Supplementary file 3 (MP4 36360 KB)

Supplementary file 4 (MP4 42804 KB)

Supplementary file 5 (MP4 36887 KB)

Supplementary file 6 (MP4 30157 KB)

Supplementary file 7 (MP4 52061 KB)

Supplementary file 8 (MP4 34073 KB)

Supplementary file 9 (MP4 48391 KB)

## Data Availability

The data will be available to other researchers by reasonable requests to the corresponding author after publication. A form of proposal with description of study objectives and statistical analysis plan will be required to make reasonability evaluation. Additional materials might be asked to provide via email. Deidentified participant data will be provided after getting approval from the corresponding author and the Seventh Medical Center of the Chinese PLA General Hospital.
