# Supplementary material for: Single-dose GC101 gene therapy for spinal muscular atrophy types II and III: an open-label single-arm study
Source: World J Pediatr. 2025 Aug 4;21(9):935–40. doi: 10.1007/s12519-025-00955-x (PMC12433340; doi:10.1007/s12519-025-00955-x)
Supplement: Supplementary file 1 — (PDF 6682 KB) [file 12519_2025_955_MOESM1_ESM.pdf]

Supplementary materials

We post it as supplied by the authors.

Supplement to: Ma XW, Jiang XY, Li ZQ, Dong XY, Ma WH, Wang YX, et al. Single-dose GC101 gene therapy for spinal muscular atrophy types II and III: an open-label single-arm study.

This supplementary material contains the following items:

1. Study design and ethics (page 2)
2. Supplementary table (page 3)
3. Supplementary figures (page 4-7)
4. Video legends (page 8)
5. Protocol (page 9-53)

Study design and ethics

This study was a single-arm, open-label clinical trial conducted in accordance with the Good Clinical Practice guidelines, the International Council on Harmonization and Declaration of Helsinki. The research protocol was approved by the Ethics Committee of the Seventh Medical Center of the Chinese PLA General Hospital (Beijing, China; S2022-001-04).

The primary objective of this study was safety assessments of GC101 injection. including adverse events (AEs), and laboratory tests of complete blood count, blood chemistry, coagulation, D-dimer test, and urinalysis at week 1, 2, 4, 6, 8, 12, 16, 28, and 52 for each patient after GC101 injection. The severity of AEs was graded according to CTCAE (Common Terminology Criteria for Adverse Events) V5.0. The secondary objective was to evaluate the efficacy of GC101. Because of the wide age range of the enrolled patients and the differences in their motor skills, different assessment methods with good applicability were selected to provide a better profile of GC101 treatment. In the study, motor function was assessed by HFMSE (Hammersmith Functional Motor Scale-Expanded) and RULM (Revised Upper Limb Module) in patients older than two years of age at week 4, 8, 12, 16, 28, and 52. Further analysis include CHOP-INTEND (Children's Hospital of Philadelphia Infant Test of Neuromuscular Disorders) in patients younger than two years of age at week 4, 8, 12, 16, 28, and 52, and 6MWT (six minute walk test) was used to assess ambulatory patients at baseline and 52 weeks after treatment. All assessments were completed by the same experienced physician. Vector genome testing was performed at week 1, 2, 4, 6, 8, 12, 16 and 52 using the sample extracted from whole blood.

Statistical analysis

The data were descriptive without statistical analysis.

Supplementary Table 1. Adverse events

Variables	All AEs	Possibly drug-related AEs	SAEs
**Any adverse events**	9	8	1
**Abnormal laboratory AEs**			
Aspartate aminotransferase increased	1	1	0
Blood CO_2 binding ability decreased	1	0	0
CK-MB increased	1	0	0
Fibrinogen decreased	1	0	0
High total cholesterol	6	6	0
High LDL cholesterol	8	8	0
High triglyceride	8	8	0
Prothrombin activity decreased	1	0	0
**Symptomatic AEs**			
Allergic rhinitis	1	0	0
Anemia	1	0	0
Back pain	1	1	0
Bronchial infection	3	0	0
Constipation	1	0	0
Cough	1	0	0
Diarrhea	1	0	0
Dyspepsia	4	0	0
Fever	3	3	0
Gastritis	1	1	0
Headache	1	1	0
Loss of appetite	1	0	0
Paronychia	1	0	0
Pneumonia	4	0	1
Productive cough	1	0	0
Submandibular gland inflammation	1	0	0
Upper respiratory infection	6	0	0
Urticaria	1	0	0
Vomiting	1	1	0

AEs were shown according to the number of patients. *AE* adverse events, *SAE* serious adverse events; *CK-MB* creatine kinase MB, *LDL* low-density lipoprotein

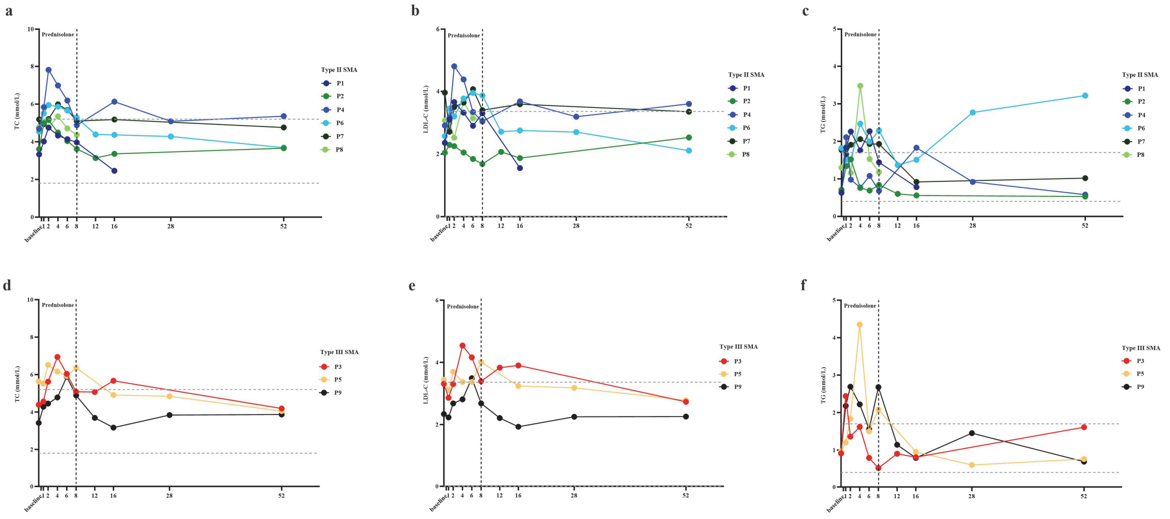

Supplementary Fig. 1 The change of total cholesterol, low density lipoprotein cholesterol, and triglyceride levels in nine patients. **a-c** Separately shows the change of total cholesterol (TC), low density lipoprotein cholesterol (LDL-C), and triglyceride (TG) levels in six type II spinal muscular atrophy (SMA) patients; **d-f** separately shows the level of TC, LDL-C, and TG levels in three type III SMA patients. The gray horizontal dashed lines represent the upper and lower level of normal range. The using period of prednisolone (the first eight week after injection) is shown by the vertical dashed line at week 8

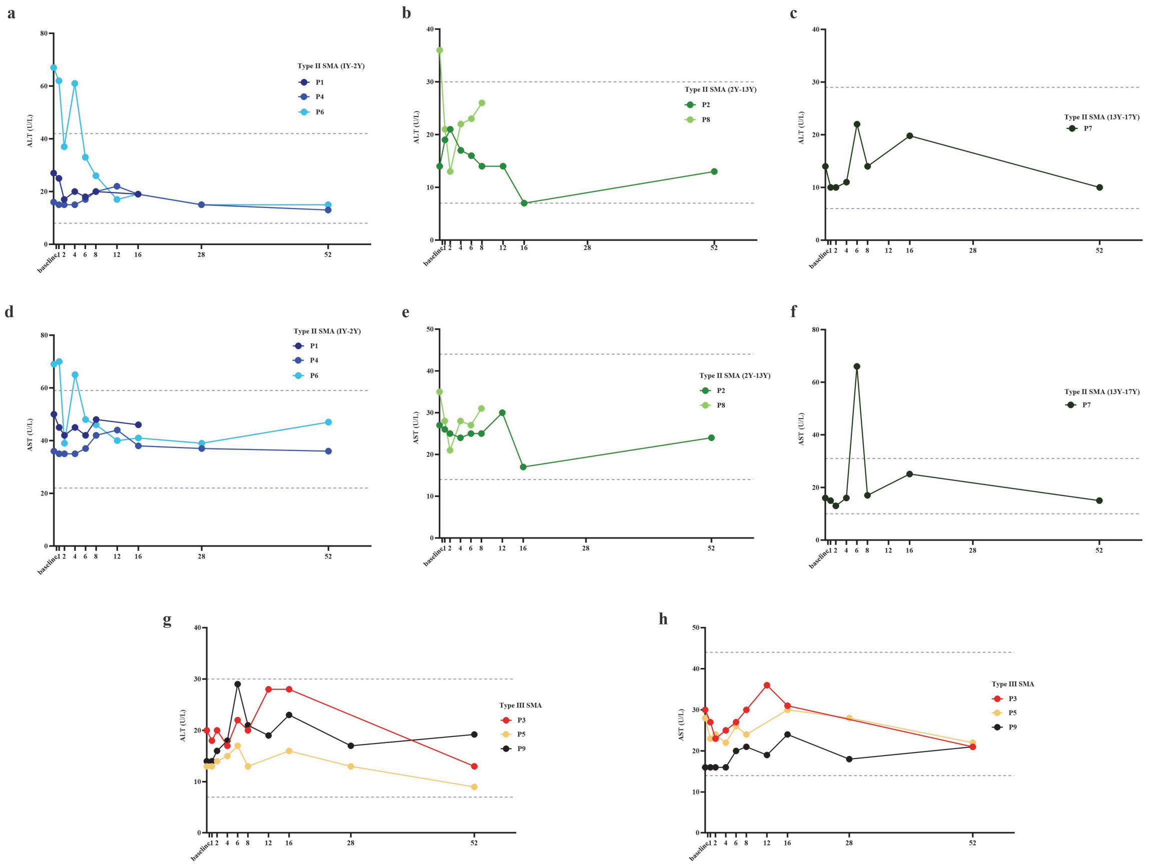

Supplementary Fig. 2 The change of alanine aminotransferase and aspartate aminotransferase levels in nine patients. **a-c** The change of alanine aminotransferase (ALT) level in six type II spinal muscular atrophy (SMA) patients grouping by different ages, respectively; **d-f** the change of aspartate aminotransferase (AST) level in six type II SMA patients grouping by different ages, respectively; **g, h** the change of ALT and AST level in three type III SMA patients. The gray horizontal dashed lines represent the upper and lower level of normal range

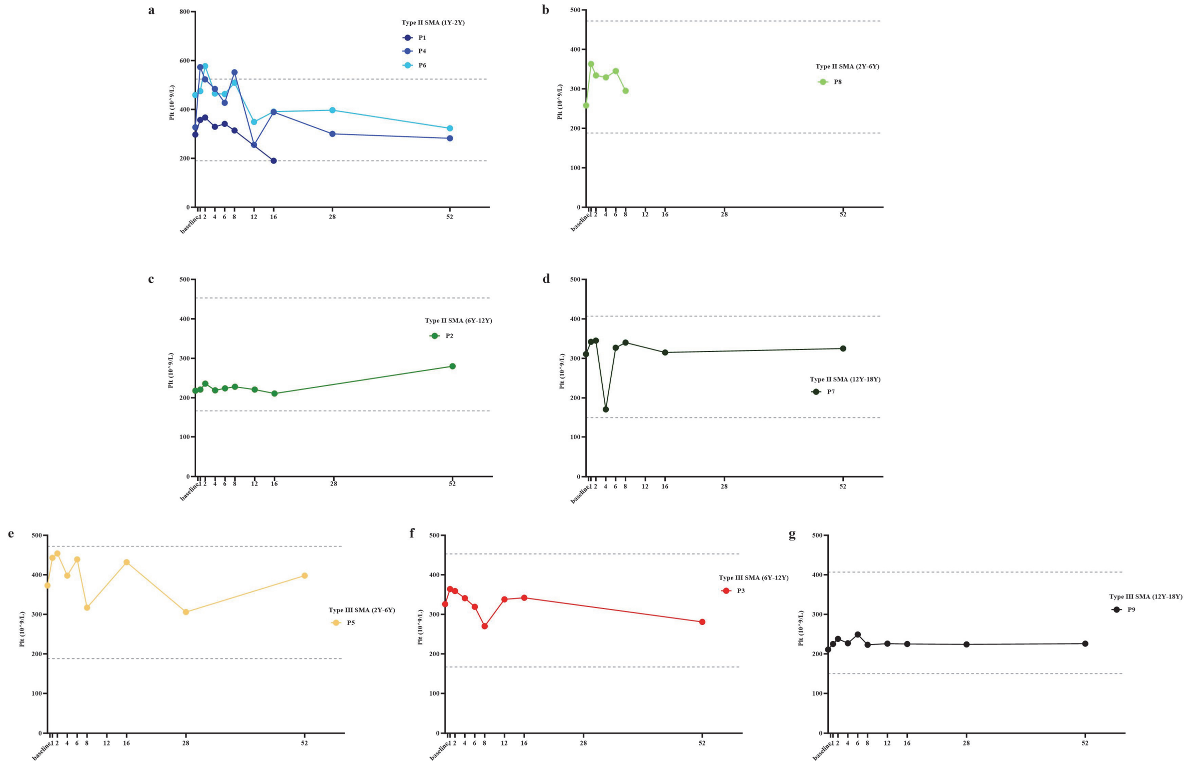

Supplementary Fig. 3 The change of platelet (Plt) level in the nine patients. **a-d** The Plt in six type II spinal muscular atrophy (SMA) patients grouping by age; **e-g** the change in three type III SMA patients. The gray horizontal dashed lines represent the upper and lower level of normal range

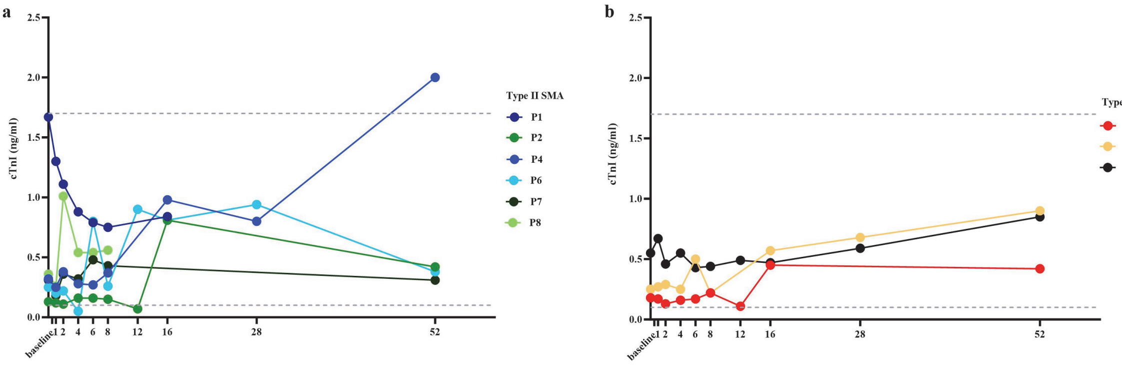

Supplementary Fig. 4 The change of cardiac troponin I (cTnI) level in the nine patients. **a** The change of cTnI level in six type II spinal muscular atrophy (SMA) patients; **b** the change in three type III SMA patients. The gray horizontal dashed lines represent the upper and lower level of normal range

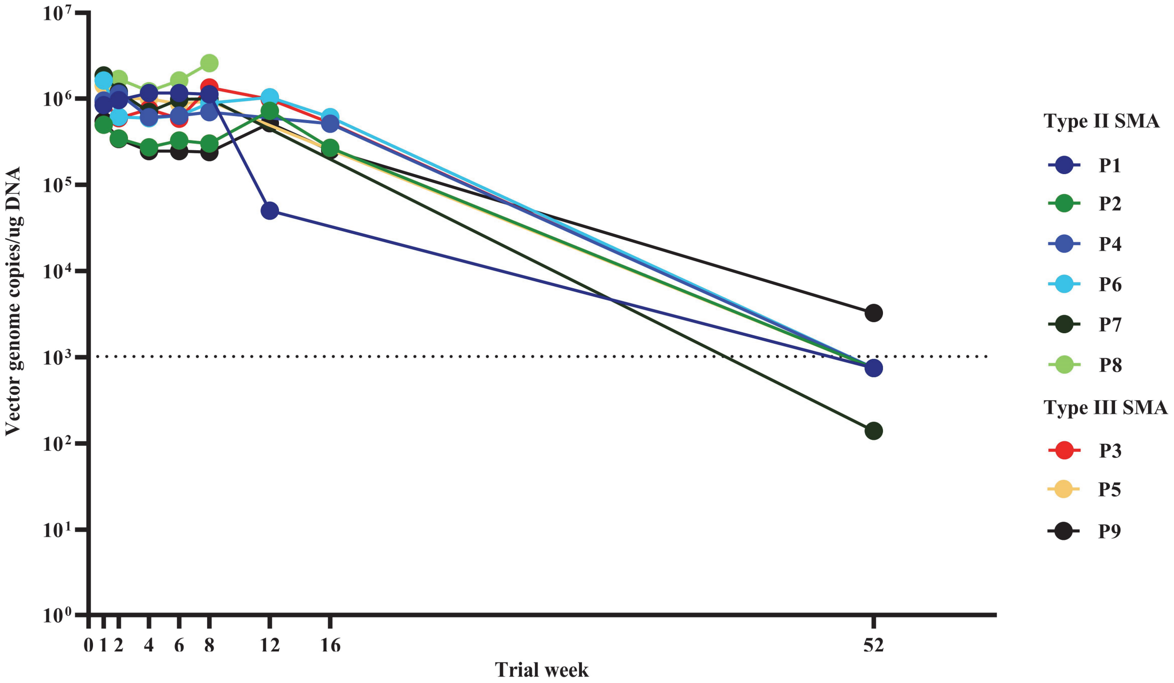

Supplementary Fig. 5 Concentration-time of the vector genome of GC101 in nine patients. The change of vector genome copies/ug DNA in six type II spinal muscular atrophy (SMA) patients and three type III SMA patients. The horizontal dotted line represents the lower limit of quantification (1000 copies/μg DNA)

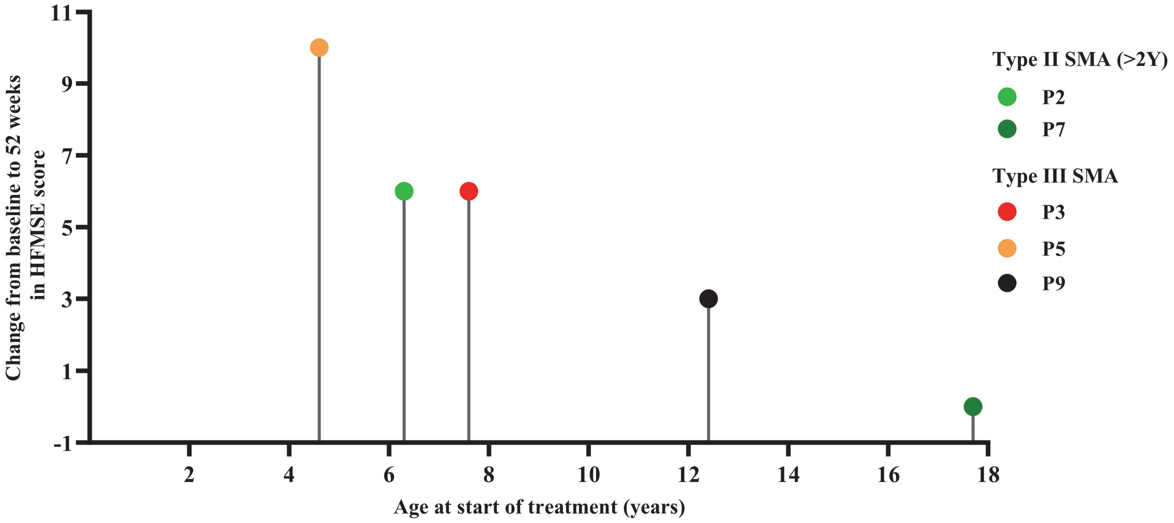

Supplementary Fig. 6 The increase of Hammersmith Functional Motor Scale-Expanded (HFMSE) score of five patients from baseline to the 52nd week of follow-up. The increase of HFMSE score from baseline to the 52nd week of follow-up in two type II spinal muscular atrophy (SMA) patients older than two years of age (excluded one patient who withdrew at week 12) and three type III SMA patients are shown by lollipop graph. X-axis shows the age when the corresponding patient received GC101 treatment

Video legends

The video showed the change of motor function before and after GC101 treatment. The time points refer to the time of taking the corresponding video, not the time that patient initially gained the ability.

Type II SMA
Video 1 (patient 1)
Before GC101 injection (1.1 years of age): cannot sit without assistance
6 weeks after GC101 injection: sit without assistance

Video 2 (patient 2)
Before GC101 injection (6.3 years of age): cannot sit stably without assistance
8 weeks after GC101 injection: sit without assistance (with hands lifting)

Video 3 (patient 4)
Before GC101 injection (1.3 years of age): sit without assistance
52 weeks after GC101 injection: walk with assistance

Video 4 (patient 6)
Before GC101 injection (0.9 years of age): cannot sit without assistance
52 weeks after GC101 injection: sit without assistance

Video 5 (patient 8)
Before GC101 injection (5.2 years of age): cannot sit without assistance
4 weeks after injection: sit without assistance

Type III SMA
Video 6 (patient 3)
Before GC101 injection (7.6 years of age): cannot stand with assistance
8 weeks after GC101 injection: stand with assistance
32 weeks after GC101 injection: stand without assistance

Video 7 (patient 5)
Before GC101 injection (4.6 years of age): walk without assistance (wadding gait), cannot jump forward (with both feet off the ground)
52 weeks after GC101 injection: walk better and faster, jump forward (with both feet off the ground)

Video 8 (patient 9)
Before GC101 injection (12.4 years of age): walk without assistance (wadding gait), jump forward (with both feet off the ground)
23 weeks after GC101 injection: jump up onto plyometric jump box
24 weeks after GC101 injection: walk better (ameliorated wadding gait)

Protocol Number: JLJY-GC101-SMA-005

Clinical study protocol

A single-arm, single-center clinical study to evaluate the safety, tolerability, and efficacy of GC101 adeno-associated virus injection in patients with type 2 and 3 spinal muscular atrophy

Study director/Responsible department: Zhi-Chun Feng/Department of Pediatrics

Sponsor: Beijing GeneCradle Technology Co., Ltd.

Testing facility: The Seventh Medical Center of the Chinese People's Liberation Army General Hospital

Study site: The Seventh Medical Center of the Chinese People's Liberation Army General Hospital

Study duration: December 2021-December 2023

Version No.: V2.0

Version date: August 10, 2022

Version revision history

Version No.	Version date	Reason for revision
V1.1	March 18, 2022	New file
V2.0	August 10, 2022	In accordance with scientific validity, feasibility, the inclusion and exclusion criteria were optimized. Considering a 20% dropout rate and a 20% misenrollment rate, an additional 4 subjects will be included, resulting in a total of 10 subjects enrolled in the study.

Sponsor signature page

We have reviewed and approved the protocol titled "A single-arm, single-center clinical study to evaluate the safety, tolerability, and efficacy of GC101 adeno-associated virus injection in patients with type 2 and 3 spinal muscular atrophy" (protocol number: JLJY-GC101-SMA-005, version number: V2.0, version date: August 10, 2022) and agree to comply with the provisions specified therein. This study can only be implemented after approval by the Ethics Committee.

We will adhere to relevant regulations and guidelines such as the current ICH-GCP, NMPA-GCP, Declaration of Helsinki, and the obligations and responsibilities of the sponsor outlined in the study protocol, and will be responsible for the quality of the investigational product used in this study.

Sponsor: Beijing GeneCradle Technology Co., Ltd.

Sponsor representative (printed): Xiao-Bing Wu

Sponsor representative (signature):

Signature date: ________________(MM/DD/YYYY)

Principal investigator signature page

We have reviewed and approved the protocol titled "A single-arm, single-center clinical study to evaluate the safety, tolerability, and efficacy of GC101 adeno-associated virus injection in patients with type 2 and 3 spinal muscular atrophy" (protocol number: JLJY-GC101-SMA-005, version number: V2.0, version date: August 10, 2022). We agree to fulfill our responsibilities in accordance with relevant regulations and guidelines such as the Declaration of Helsinki, ICH-GCP, NMPA-GCP, and the provisions specified in this study protocol. This study can only be implemented after approval by the Ethics Committee.

We will adhere to relevant regulations and guidelines such as the current ICH GCP, NMPA GCP, the Declaration of Helsinki, and the obligations and responsibilities of investigators outlined in the study protocol. Modifications to the protocol can only be implemented after approval by the sponsor and re-approval or approval for filing by the ethics committee, unless measures must be taken to protect the safety, rights, and interests of subjects.

I will keep this protocol and related contents confidential.

Testing facility: The Seventh Medical Center of the Chinese People's Liberation Army General Hospital

Principal investigator (printed): Zhi-Chun Feng

Principal investigator (signature):

Signature date: ________________(MM/DD/YYYY)

Test institution signature page

We have reviewed the protocol titled "A single-arm, single-center clinical study to evaluate the safety, tolerability, and efficacy of GC101 adeno-associated virus injection in patients with type 2 and 3 spinal muscular atrophy" (protocol number: JLJY-GC101-SMA-005, version number: V2.0, version date: August 10, 2022) and agree to comply with the provisions listed therein. This study can only be implemented after approval by the Ethics Committee.

We will adhere to relevant regulations and guidelines such as the current ICH GCP, NMPA GCP, Declaration of Helsinki, and various documents established with the sponsor, and strictly implement the clinical study protocol in accordance with the provisions.

I will keep this protocol and related contents confidential.

Sample testing institution:

Project leader (printed):

Project leader (signature):

Signature date: ________________(MM/DD/YYYY)

Signature page of statistical and data management institution

We have reviewed the protocol titled "A single-arm, single-center clinical study to evaluate the safety, tolerability, and efficacy of GC101 adeno-associated virus injection in patients with type 2 and 3 spinal muscular atrophy" (protocol number: JLJY-GC101-SMA-005, version number: V2.0, version date: August 10, 2022) and agree to comply with the provisions listed therein. This study can only be implemented after approval by the Ethics Committee.

We will adhere to relevant regulations and guidelines such as the current ICH-GCP, NMPA-GCP, Declaration of Helsinki, and the obligations and responsibilities of statistical and data management institution specified in the clinical study protocol.

I will keep this protocol and related contents confidential

Data statistics institution: The Seventh Medical Center of the Chinese People's Liberation Army General Hospital

Head of statistical and data management (printed):

Head of statistics and data management (signature):

Signature date: ________________(MM/DD/YYYY)

List of abbreviations

SMA spinal muscular atrophy

SMN1 survival motor neuron 1

SMN2 survival motor neuron 2

AAV adeno-associated virus

CRF case report form

SOP standard operating procedure

ADR adverse drug reaction

ULN upper limit of normal

Protocol synopsis

**Study title**	A single-arm, single-center clinical study to evaluate the safety, tolerability, and efficacy of GC101 adeno-associated virus injection in patients with type 2 and 3 spinal muscular atrophy
**Study objectives**	**Primary objective:**<br>To evaluate the clinical safety and tolerability of a single dose of GC101 adeno-associated virus injection (hereinafter referred to as GC101) for the treatment of subjects with type 2 and 3 spinal muscular atrophy (SMA).<br>**Secondary objective:**<br>To evaluate the short-term and long-term efficacy of a single dose of GC101 in the treatment of subjects with type 2 and 3 SMA.
**Study design**	This is an investigator-initiated clinical study for the treatment of type 2 and 3 SMA with a single dose of GC101.<br>**Study design**<br>This study adopts a single-arm, single-center, open-label, single-dose design, with one dose group, enrolling a total of 10 subjects. The study dose is set at 1.2E+14 vector genomes (vg)/person.<br>Using a sentinel study design, the first subject in the test group serves as the sentinel. After receiving a single dose of the investigational product, the subject undergoes a safety assessment for at least 4 weeks before subsequent subjects are allowed to receive the drug. The dosing interval for subsequent subjects should be ≥ 24 hours.<br>Study termination criteria:<br>According to the CTCAE 5.0 criteria, more than half of the subjects experience Grade 2 or higher drug-related adverse events (AEs), or more than one-third of the subjects experienced Grade 3 or higher drug-related AEs, or there is one subject experiencing a drug-related serious adverse event (SAE).<br>During the study period, the motor-neuromuscular function of the subjects will be evaluated, and the clinical symptom changes of subjects after medication will be regularly observed.
**Total number of subjects**	10
**Eligibility criteria**	**Inclusion criteria**<br>Subjects must meet all of the following criteria to be eligible for inclusion in this study.<br>1. During the screening process, subjects should be aged between 6 months and 18 years (excluding 18 years old), with no gender restrictions.<br>2. Clinical diagnosis of type 2 and type 3 5q SMA;<br>3. Type 2 patients lack the ability to walk, while type 3 patients either lack the ability to walk or possess the ability to walk but are unable to squat and stand up independently.<br>4. Possessing independent sitting ability (defined as: being able to sit independently for more than 5 seconds with the support of one's own upper limbs).<br>5. One or both hands can only be raised to the height of the mouth;<br>6. Negative for SMN2 gene modification mutation (c.859 G>C);<br>7. The subjects or their guardians are able to understand and are willing to comply with the requirements and procedures of the study protocol, voluntarily participate, and sign the informed consent form.
	**Exclusion criteria**<br>Individuals meeting any of the following criteria will be ineligible to participate in this study.<br>1. Having participated in any other gene therapy clinical studies.<br>2. Unable to participate in the late-stage follow-up of the study.<br>3. Within 30 days prior to the screening period, the use of SMN2-targeted modulating drugs (Spinraza or Risdiplam) has occurred.<br>4. Serum anti-AAV9 neutralizing antibody titer >1:10 (if serum anti-AAV9 neutralizing antibody titer > 1:10, then cerebrospinal fluid anti-AAV9 neutralizing antibody titer will be measured. If cerebrospinal fluid anti-AAV9 neutralizing antibody titer > 1:10, the subject will be ineligible for this study).<br>5. Patients with a predisposition to allergies, including those with hypersensitivity or allergic reactions to prednisolone or other corticosteroids, or their excipients, as well as those with hypersensitivity or allergic reactions to iodine or iodine-containing products.<br>6. Contraindications to lumbar puncture or intrathecal therapy are present.<br>7. X-ray examination reveals severe scoliosis of the spine (≥ 50°).<br>8. Planned or anticipated spinal scoliosis correction surgery within 1 year after administration.<br>9. Invasive ventilation support or blood oxygen saturation < 95%.<br>10. Severe non-respiratory diseases requiring systemic treatment and/or hospitalization within two weeks prior to the initiation of the study.<br>11. Occurrence of respiratory infections within four weeks prior to the study that require medical care, medical intervention, or any increase in supportive care.<br>12. Occurrence of severe non-pulmonary/respiratory infections or concomitant diseases within four weeks prior to the administration of the study drug, which, in the opinion of the principal investigator (PI), may pose unnecessary risks to gene transfer, such as significant renal or liver impairment, known epilepsy, diabetes, idiopathic hypocalciuria, or systemic cardiomyopathy.<br>13. History of bacterial meningitis or brain or spinal cord diseases, including tumors, or magnetic resonance imaging (MRI) or computed tomography (CT) abnormalities that may interfere with lumbar puncture (LP) or cerebrospinal fluid circulation.

	14. Unable to discontinue the use of laxatives or diuretics within 24 hours prior to drug administration.<br>15. Any serious or active disease other than SMA, which the investigator believes may interfere with the subject's treatment, evaluation, or compliance with the study protocol. Examples include cardiovascular diseases, kidney diseases, liver diseases, endocrine disorders, malignant tumors, diabetes, hypersensitivity, infectious diseases, coagulation abnormalities, immunodeficiency disorders, and severe mental and neurological disorders.<br>16. Human immunodeficiency virus (HIV) antibody-positive, or hepatitis B surface antigen-positive, or hepatitis C antibody-positive, or treponema pallidum (syphilis) antibody-positive.<br>17. History of alcohol consumption and drug abuse.<br>18. The investigator deems it inappropriate for the subject to be enrolled in this study.
**Administration**	1. Prior to injection, warm the investigational product to room temperature and quickly thaw it at 37 °C. The drug should melt within 5 minutes and appear as a colorless, transparent to opalescent liquid. If particulate precipitation occurs, do not use the drug.<br>2. After thawing, the drug must be used within 1 hour. If delayed use is required, it should be stored at 2-8 °C and used within 7 days.<br>3. Adjust the injection volume to 2 mL using sterile physiological saline for injection, following aseptic procedures.<br>4. Perform intrathecal injection according to standard operating procedures.<br>5. After completing the injection, press the injection site for 5 minutes. If there is still leakage, continue pressing.<br>6. The subject rested in supine position.
**Efficacy and safety evaluation**	Efficacy evaluation measures (primary and secondary efficacy endpoints)<br>1. Primary efficacy measures:<br>1) Changes in the Hammersmith Functional Motor Scale Expanded (HFMSE) score within 52 weeks of treatment compared to baseline.<br>2) Changes in the Revised Upper Limb Module (RULM) score for neuromuscular function within 52 weeks of treatment compared to baseline.<br>2. Secondary efficacy measures:<br>1) Changes in muscle MRI compared to baseline within 52 weeks of treatment.<br>2) Changes in muscular MUNE within 52 weeks of treatment compared to baseline.
	Safety evaluation measures<br>1. AE/SAE.<br>2. Vital signs: blood pressure, pulse, respiration, and body temperature.<br>3. Physical examination signs.<br>4. Laboratory examinations: hematology, urinalysis, blood chemistry, coagulation function, and viral testing.<br>5. 12-lead electrocardiogram (ECG).<br>6. Echocardiography.<br>7. PK parameters derived from blood drug concentration (viral load)-time data.<br>8. Generation and variation patterns of drug-resistant antibodies (anti-AAV9 and anti-SMN).
**Statistical methods**	**General principles**<br>Quantitative data are generally described using mean, median, standard deviation, maximum, and minimum values, while count data or ordinal data are described using frequency and frequency distribution.<br>**Safety analysis**<br>AEs will be coded using MedDRA and classified into two levels: System Organ Classification (SOC) and Preferred Term (PT). This study primarily focuses on the statistical analysis of Treatment-Emergent Adverse Events (TEAEs) occurring after drug administration, while AEs occurring before drug administration will be listed. The occurrence, number of cases, and incidence of all AEs, drug-related AEs, SAEs, and AEs leading to discontinuation will be calculated. The severity of AEs and their relationship to the study drug will be described in tabular form. Separate lists will be provided for drug-related AEs, non-drug-related AEs, AEs leading to discontinuation, and SAEs.<br>A statistical description of the changes in vital sign parameters from baseline to various time points after treatment will be provided, respectively, including the number of subjects, mean, standard deviation, median, minimum, and maximum values.<br>The changes in laboratory tests, physical examinations, and 12-lead electrocardiograms from baseline to each time point after dosing in the form of a cross-tabulation before and after administration will be listed. Abnormal laboratory tests, physical examinations, and 12-lead electrocardiogram examinations at each time point after dosing will be listed in a tabular format.<br>**Efficacy analysis**<br>The changes in neuromuscular function scores at each evaluation time point compared to baseline in the study subjects will be calculated.<br>**Pharmacokinetics (viral load)**<br>Using the non-compartmental analysis method of Phoenix WinNonlin software, the pharmacokinetic (PK) parameters of GC101 injection will be analyzed, including AUC, C_{max}, CL, $T_{1/2}$, etc.<br>**Immunogenicity**<br>The incidence and occurrence time of binding antibodies and neutralizing antibodies will be determined, antibody titers will be measured, and the proportion of positive antibodies will be calculated.

**Study Duration**	52 weeks

I. Study background

1. Value of topic selection

GC101 is a gene therapy drug developed for spinal muscular atrophy (SMA), a currently incurable condition in China. The aim of the product is to provide a long-lasting cure for SMA through a single intrathecal injection, addressing the unmet clinical needs of SMA patients.

According to incomplete statistics, the survival condition of SMA patients in China is quite difficult. The rarity of the disease in the general population, the lack of understanding of various clinical features by physicians, and the limitations of available diagnostic tools (histopathology and genetic screening) contribute to a high misdiagnosis rate and poor prognosis for patients. Delayed diagnosis is a major barrier to optimizing management for SMA patients in China, with the time from the first appearance of clinical symptoms to diagnosis often spanning several years. By the time of diagnosis, SMA patients have often missed the golden window for treatment and are frequently wheelchair-bound, resulting in a significant impact on their lives. In addition, the cost of treatment is extremely high. Given the significant unmet medical needs for this rare and fatal disease, there is an urgent need for clinical drug development for SMA to facilitate timely access to effective treatments for the patient population.

2. Disease introduction

SMA is an autosomal recessive genetic disorder caused by degeneration of the lower motor neurons in the spinal cord and somatic motor nuclei in the brainstem. Patients with SMA exhibit symmetrical progressive muscle atrophy and weakness, starting from the proximal end and eventually affecting all skeletal muscles. SMA is one of the leading genetic causes of infant and child mortality and has been included in the "first list of rare diseases" jointly issued by the National Health and Wellness Commission and other five departments. SMA can be divided into 5q SMA and non-5q SMA, with 5q SMA accounting for 95% of cases. Based on the severity and age of onset, SMA is internationally classified into four types and eight subtypes (see Table 1).

Table. 1 SMA classification and characteristics

Classification	Age of onset	Sport milestones	Clinical presentation	Natural course	SMN2 copy number
0	Before or at birth	None	Except for the eyeballs, there is almost no activity in the limbs, trunk, and face, and no sucking action; congenital joint contractures, muscle atrophy, and disappearance of reflexes; mechanical ventilation is required immediately after birth; congenital heart disease may be present as a comorbidity	Months	1
1	< 6 mon	Not able to sit independently	Infants with soft muscle tone, severe hypotonia, limb weakness; tongue, facial, and masticatory muscle weakness; bell-shaped thorax; prone to recurrent respiratory infections and respiratory failure	Group 1a and 1b: ≤ 2 y; Group 1c: 2-y survival probability is 95%	1a primarily refers to 1; 1b primarily refers to 2; 1c primarily refers to 3
2	6-18 mon	Able to sit independently, but unable to walk independently.	During infancy, there is a gradual worsening of generalized muscle weakness and hypotonia; delayed motor development, tongue muscle fibrillation or hand muscle fasciculation; may be accompanied by joint contractures and scoliosis, affecting respiratory function	Most survive to adulthood	Mainly 3
3	> 18 mon to 10 y	Able to walk independently	Within the first year of life, motor development is normal; during childhood, progressive proximal muscle weakness occurs, with the lower limbs being more affected than the upper limbs. As the disease progresses, the ability to walk is lost; muscle fasciculations can be observed. In later stages, manifestations such as scoliosis, joint deformities, and respiratory insufficiency may appear	Lifespan does not shorten or mildly decreases	3 or 4
4	Adult	Able to run and jump	The onset of the disease occurs during adolescence or adulthood, characterized by proximal limb weakness starting in the lower extremities, with a slow progression of the condition	Life expectancy is generally unaffected	Mainly 4

Types 2 and 3 SMA, also known as Kugelberg-Welander, typically manifest in patients after 6 months of age. These patients can initially acquire independent walking abilities; however, as the disease progresses, some may lose their walking abilities during childhood, adolescence, or adulthood. Patients who exhibit symptoms before the age of 3 are classified as type 3A SMA, while those who develop symptoms after the age of 3 are classified as type 3B SMA. Approximately 50% of Type 3A SMA patients lose their ability to walk independently around the age of 14, with a higher degree of disability compared to type 3B. Patients with types 2 and 3 SMA often present with proximal muscle weakness, with the legs being more affected than the arms, and tendon reflexes still present. Over time, proximal reflexes disappear, and tremors, as seen in type 2 SMA patients, may also manifest. Compared to the general population, the life expectancy of patients with types 2 and 3 SMA does not show significant differences.

The incidence of SMA is approximately 1 in 6000 to 1 in 10,000, with a population carrier rate of 1 in 35 to 1 in 60. There remain unmet clinical needs in the medical management of this condition.

3. Current treatment status of SMA

Before 2016, there were no specific treatment drugs for SMA. From 2016 to the present, three targeted drugs for SMA have been approved worldwide: Spinraza (antisense oligonucleotide), Zolgensma (AAV), and Risdiplam (small molecule). Currently, the available drugs for treating SMA in China are Spinraza and Risdiplam.

Spinraza and Risdiplam are both therapeutic drugs targeting SMN2 splicing regulation. Spinraza is an antisense oligonucleotide drug, while Risdiplam is a nucleotide analog. They work by interacting with SMN2 and its precursor transcript (pre-mRNA), altering the original splicing pattern of SMN2 pre-mRNA, increasing the inclusion of exon 7, and enhancing SMN protein levels. Clinical studies have shown that Spinraza has a clear

therapeutic effect and good safety profile. However, due to the need for lifelong administration and the high cost of the drug, very few patients in China can afford it. Moreover, repeated administration increases the risk of intrathecal injections, and long-term use may lead to drug tolerance and resistance. Risdiplam also requires daily oral administration for life, and its efficacy is limited.

Zolgensma is a gene replacement therapy that involves a single intravenous injection of recombinant adeno-associated virus into the body. By utilizing the scAAV9 viral vector, the SMN1 gene is delivered directly to the central nervous system through the blood-brain barrier, increasing the expression of SMN protein in the body. The biodistribution of the drug meets the requirements of the disease's biodistribution. Furthermore, AAV vector-mediated gene transfer can provide long-lasting and effective treatment for SMA, achieving long-term benefits with a single administration. The announced price for Zolgensma is $2.125 million per dose, and it has not yet been marketed in China.

In summary, the current treatment methods and drug prices in the domestic market are unable to meet the medical needs of patients with SMA. Gene therapy can provide a one-time, comprehensive treatment option for patients with rare genetic diseases such as SMA. GC101 injection is an ideal therapeutic drug for SMA.

4. Introduction to GC101 adeno-associated virus injection

GC101 adeno-associated virus injection (hereinafter referred to as GC101) is a gene therapy drug developed independently by Beijing GeneCradle Technology Co., Ltd. for patients with 5q SMA. The active ingredient of GC101 is a non-replicating adeno-associated virus vector (AAV9), which can be used to treat SMA caused by SMN1 gene deletion. GC101 is a gene therapy biological product, administered through a single intrathecal injection, and the expressed human SMN protein can fundamentally treat SMA.

GC101 has undergone non-clinical pharmacological and toxicological studies in rodents and non-human primates (cynomolgus macaques). The efficacy, safety, and toxicology of the product have been evaluated in SMA animal models. The results of all tests indicate that GC101 is safe and effective, with a clear dose-dependent relationship. Safety studies show that the test animals have a good tolerance to GC101. Based on the efficacy and safety conclusions of GC101, it is proven that GC101 is suitable for gene therapy in patients with 5q SMA (including types 1, 2, and 3). The maximum tolerated dose of the product in test animals is > 1.9E+14 vg/kg. GC101 is a clarified, colorless, or opalescent sterile injectable solution, containing 1.2E+14 vg of serotype 9 recombinant adeno-associated virus particles per milliliter. The solution has a pH of ~7.3 and an osmotic pressure of 285-310 mOsmol/kg. The GC101 formulation is filled into 2 mL sterile borosilicate glass vials (pre-washed, pyrogen-free) with a labeled fill volume of 2 mL. The vials are sealed with a chlorobutyl rubber stopper coated with a sterile polyethylene-tetrafluoroethylene film and an aluminum-plastic combination cap for antibiotic vials. The product is stored at -70 °C or below.

After the formulation has passed the quality tests for sterility and endotoxins, it is permitted for use in the study.

5. Nonclinical studies

5.1 Efficacy studies

The collaborating study team associated with this protocol (Beijing GeneCradle Technology Co., Ltd.) has developed the gene therapy drug, "GC101", which utilizes adeno-associated virus serotype 9 (AAV9) to carry the human SMN protein-encoding gene expression cassette. The drug is delivered to the central nervous system

through a single intrathecal injection, resulting in continuous expression of the human SMN protein. Preclinical studies have shown that after a single lateral ventricular injection of GC101, the median survival time of severe SMA model mice is increased, their appearance is improved, and they exhibit near-normal motor abilities. Pathological results demonstrate an increase in the number of spinal cord anterior horn motor neurons and fuller cell morphology, indicating that the underlying cause of SMA has been effectively corrected. It is predicted that the therapeutic effect of GC101 may persist for an extended period in clinical settings.

The dose-effect relationship study of GC101 in the treatment of severe SMA model mice demonstrated that after GC101 treatment intervention, significant improvements were observed in the appearance characteristics, behavioral studies, and survival of severe SMA. Compared to the untreated group, the median survival of severe SMA model mice significantly increased after administration. When the injection dose increased from 4.4E+13 vg/kg to 8.0E+13 vg/kg, a noticeable jump in the longest survival time appeared in the survival curve, with approximately 43% of mice showing long-term effectiveness of GC101 treatment. When the injection dose increased from 8.0E+13 vg/kg to 1.6E+14 vg/kg, the overall survival curve showed significant improvement, with the median survival of more than 80% of the model mice exceeding 30 days, and more than 60% of the model mice surviving over 60 days, indicating that the therapeutic effect of GC101 is significant and stable.

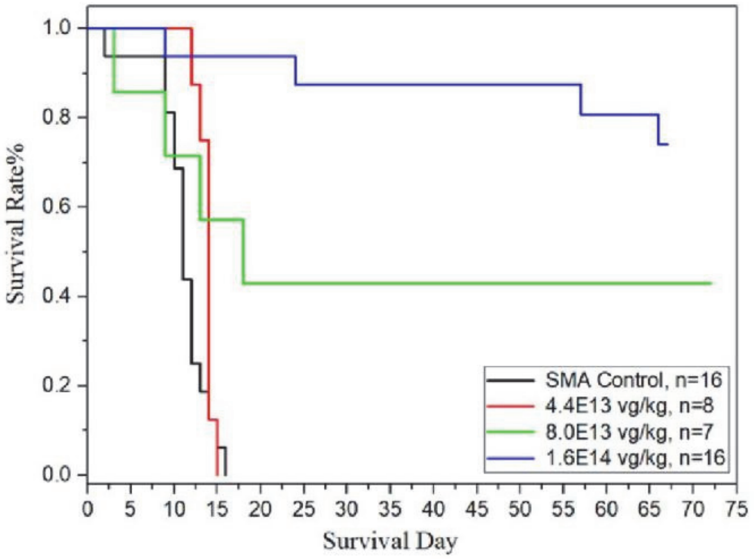

Fig. 1 Median survival curve in severe SMA model treated with single intracerebroventricular administration of GC101

The therapeutic study of GC101 in a mild SMA mouse model demonstrated that a single intracerebroventricular injection significantly increased the number of spinal cord anterior horn motor neurons in the model mice. The tail length of the model mice was largely preserved, and the mice survived for an extended period (close to their entire lifespan). Treatment of type 2 and 3 SMA model mice showed efficacy within the dose range of 2.7E+13 vg/kg to 1.6E+14 vg/kg, with a decreasing trend in efficacy observed at the 2.7E+13 vg/kg dose.

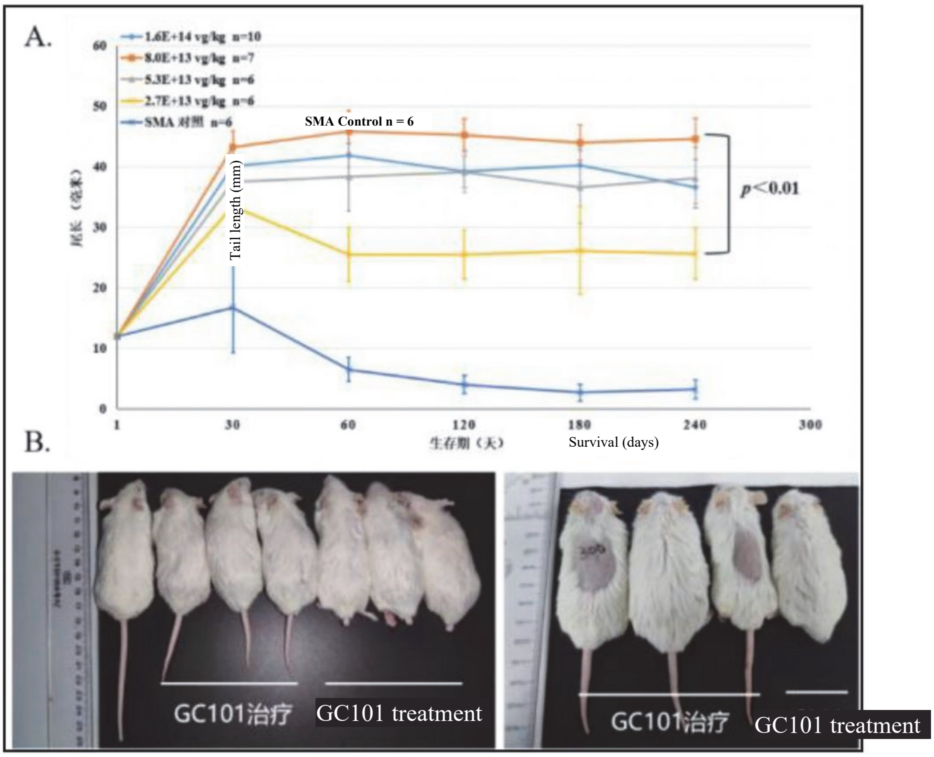

Fig. 2 Long-term efficacy data of single intracerebroventricular injection of GC101 in treating SMA 3 model. **A** Time-dependent curve of tail length in SMA 3 mice; **B** general appearance of the 8.0E+13 vg/kg dose group approximately one year after administration

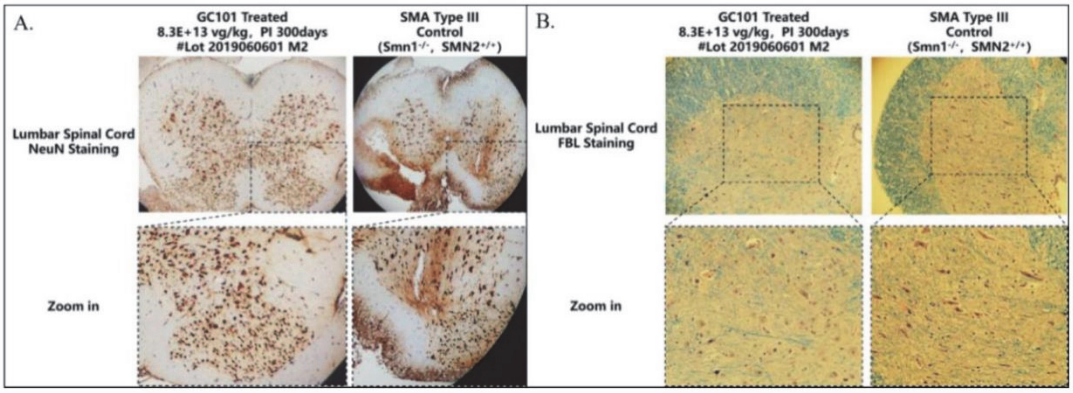

Fig. 3 Spinal cord pathology of SMA3 mice treated with a single intracerebroventricular injection of GC101. **A** NeuN-stained transverse view of the lumbar spinal cord in mice; **B** FBL-stained transverse view of the lumbar spinal cord in mice

5.2 Safety studies

Table 2. Single-dose toxicity study

Study number	Study name	Study number
Study 1	Preliminary intravenous injection toxicity study of RDGT-002 in neonatal mice	TS18078-YX
Study 2	Toxicity study of GC101 by intracerebroventricular injection in mice	GC-SMP-08-PR07（GC101）
Study 3	Toxicity study of GC101 by intravenous injection in mice	GC-SMP-08-PR05（GC101）
Study 4	Exploratory single-dose toxicity study of GC101 by intravenous injection in C57BL/6N adult mouse	GC-SMP-08-PA16（GC101）
Study 5	Expanded single-dose toxicity study of GC101 by intracerebroventricular injection in C57BL/6N neonatal mice	SN20274-XJ
Study 6	Preliminary single-dose toxicity study of GC101 in juvenile cynomolgus macaques	TS19090-HC
Study 7	Toxicity study of GC101 by intrathecal injection in cynomolgus macaques	P21-073-SD

Study 1: Preliminary intravenous injection toxicity study of RDGT-002 in neonatal mice

In this study, 26 neonatal C57BL/6N mice (aged 48-60 hours post-birth) were administered different doses of RDGT-002 product (structurally identical to GC101 drug, produced under a non-GMP triple-plasmid process) via superficial temporal vein injection to preliminarily investigate whether high-dose intravenous administration would result in peripheral toxicity. The results demonstrated that the product has good safety, and even with high-dose systemic delivery, no significant toxicity was observed in peripheral organs. The maximum tolerated dose (MTD) in this study was 4E+14 vg/kg.

Study 2: Intracerebroventricular injection toxicity study of GC101 in mice

The study involved intracerebroventricular injections of doses 5-10 times higher than the intended clinical dose (1.6E+14 vg/kg and 5.47E+14 vg/kg, in neonatal mice). No toxic reactions or animal deaths were observed during cage-side monitoring, and the body weight of mice in each group increased steadily during the observation period. No abnormalities were observed in the organs and tissues of the animals in each group. The MTD was 5.47E+14 vg/kg. Pathological examination results showed that after a single intracerebroventricular intervention with GC101 for 4 weeks, no significant pathological changes were observed in the nervous system tissues and most peripheral tissues and organs. However, small focal necrosis was observed in some areas of the liver in the treated animals, suggesting that GC101 has potential hepatotoxicity risk at higher doses. This finding is consistent with the toxicity study results of similar products internationally.

Study 3: Intravenous injection toxicity study of GC101 in mice

In order to maximize the exposure of GC101's toxic effects in peripheral tissues, a single intravenous study was conducted in wild-type C57BL/6N mice (significantly higher than the intended clinical dose in this study protocol). No mortality was observed in mice after a single injection, and histopathological examination did not reveal any severe drug-related tissue toxicities. Mild inflammatory changes were only observed in the liver tissue. These inflammatory changes are similar to the preclinical toxicity study results of the approved drug Zolgensma, which is considered a transient T-cell response to the AAV vector, resulting in transient hepatitis. This study indicates that GC101 has a good safety profile, with low adverse risks that are expected to be manageable and reversible. The MTD for this study is 3E+14 vg/kg.

Study 4: Exploratory single-dose toxicity study of GC101 by intravenous injection in adult C57BL/6N Mice

In this study, four adult C57BL/6N mice were intravenously injected with a dose of 5.3E+13 vg/kg of GC101 to investigate whether a high dose of intravenous injection would cause toxicity to the peripheral system. The results demonstrated that the product has good safety, with no observed pathological changes related to the test article. The No Observed Adverse Effect Level (NOAEL) for this study was determined to be 5.3E+13 vg/kg.

Study 5: Expanded single-dose toxicity study of GC101 by intracerebroventricular injection in C57BL/6N neonatal mice

Under GLP conditions, an expanded single-dose toxicity study of intracerebroventricular injection of GC101 in neonatal mice was conducted to evaluate the safety of the drug. A total of 130 neonatal C57BL/6N mice (aged 24-48 hours) were administered a single intracerebroventricular injection of GC101 at doses of 5.3E+13 vg/kg, 1.2E+14 vg/kg, and 4E+14 vg/kg. All injected animals exhibited normal weight gain and did not show any significant toxic reactions or mortality, indicating good tolerability at these doses (MDT > 4E+14 vg/kg). Histopathological examination at 2 and 6 weeks post-dose revealed a small amount of inflammatory cell infiltration in the liver of the high-dose group, which resolved by 13 weeks post-administration. Overall, the results demonstrate that the safety profile of GC101 injection is favorable. No test-article-related pathological changes were observed, and the NOAEL and MTD for this study were determined to be 4E+14 vg/kg.

Study 6: Preliminary single-dose toxicity study of GC101 in juvenile cynomolgus macaques

A single intrathecal injection toxicity pre-study was conducted on three juvenile cynomolgus macaques (aged 5 months), with a dose of 3.28E+13 vg/animal. The results showed that the general condition of the animals was good, with no significant acute toxic reactions or drug-induced deaths, no histopathological changes, and no tumor occurrence. Histopathological examination revealed no test article-related toxic reactions. The overall study results indicate that GC101 has good safety in non-rodent animals. The NOAEL for this study is 3.28E+13 vg/animal.

Study 7: Single-dose toxicity study of GC101 by intrathecal injection in cynomolgus macaques

Under GLP conditions, a single intrathecal injection of GC101 was administered to 12 cynomolgus macaques (aged 1.5 years) in a toxicity study, with a dose of 9.6E+13 vg/animal. The general condition, body weight changes, clinical pathology, hematology, coagulation, and other measures of the macaques were dynamically monitored after administration, and anatomical and histopathological analyses were performed at the end of the observation period. All injected macaques exhibited normal weight gain, with no significant toxic reactions or deaths observed. During the 29 days post-administration, the results of the dynamic monitoring subjects showed no differences compared to the control group or pre-treatment. Histopathological examination of the tissues collected on day 29 post-administration revealed no test article-related toxic reactions. These results indicate that GC101 has good tolerability and safety at this dose. The NOAEL for this study is 9.6E+13 vg/animal.

II. Study objectives

1. Primary objective

To evaluate the clinical safety and tolerability of a single dose of GC101 in subjects with type 2 and 3 SMA.

2. Secondary objective

To evaluate the short-term and long-term efficacy of single dose of GC101 in treating subjects with type 2 and 3 SMA.

III. Study design type, principles, and study procedures

1. Study design

This study adopts a single-arm, single-center, open-label, single-dose design, with one dose group, enrolling a total of 10 subjects. The study dose is 1.2E+14 vg/person.

Using a sentinel study design, the first subject in the test group serves as the sentinel. After receiving a single administration of the investigational product, the subject undergoes a safety assessment for at least 4 weeks before the subsequent two subjects are allowed to receive the investigational product.

Route of administration/dosing frequency: single intrathecal injection.

Dose: 1.2E+14 vg/person

Study population: patients aged 6 months to 18 years (excluding 18 years) with type 2 and type 3 SMA

2. Rationale for dose selection

Preclinical studies have shown that a dose range of 2.7E+13 vg/kg to 1.6E+14 vg/kg has significant therapeutic effects on mild SMA mouse models, with an optimal efficacy at 8.0E+13 vg/kg. Due to the limitations of the disease progression window in test animal models, interventions must be performed in newborns. Therefore, nonclinical study data can be used to extrapolate the intended clinical dose for humans based on body weight per kilogram. The body weight of a one-month-old infant is approximately 4 kg, so the intended clinical dose range for newborns, derived from the test animal dose range of 2.7E+13 vg/kg to 1.6E+14 vg/kg, is calculated to be 1.08E+14 vg/person to 6.4E+14 vg/person.

Study observations indicate that during the developmental process of ages 0-3, the spinal cord volume gradually develops to a stable state between 2-3 years of age. Therefore, in children aged 3 and above, the spinal cord volume or the number of spinal motor neurons will not significantly change with increasing age. As a result, the age range of subjects enrolled in this study does not require adjustment of the medication dosage based on body weight.

Based on the non-clinical pharmacodynamic (PD) data, it can be inferred that the effective dose range of GC101 injection for subjects aged 6 months to 25 years with type 2 and 3 SMA is between 1.08E+14 vg/person and 6.4E+14 vg/person.

In the preclinical non-human primate toxicity study, the maximum dosing method was used to investigate the potential toxicity of GC101, with a dose of 9.6E+13 vg/animal (3.84E+13 vg/kg). The toxicity evaluation results at this dose were: no test article-related toxic reactions, i.e., NOAEL. The safety conclusion supports the intended clinical dosing.

Referring to the international clinical experience of similar products, the doses for intrathecal administration in the Zolgensma clinical study (NCT03381729) are 6.0E+13 vg/person, 1.2E+14 vg/person, and 2.4E+14 vg/person. The dose selection for GC101 is consistent with international clinical experience, ensuring high reliability.

Based on the comprehensive non-clinical pharmacology and toxicology study results of GC101, as well as the clinical experience analysis of similar international products, we believe that the study dose of 1.2E+14 vg/person can be applied in this study.

3. Dose selection for response

Based on the preclinical efficacy study data, the calculated clinical dose for use is theoretically effective in improving motor function in subjects with type 2 and 3 SMA.

4. Termination criteria

According to the CTCAE 5.0 criteria, more than half of the subjects experience Grade 2 or higher drug-related AEs, or more than one-third of the subjects experienced Grade 3 or higher drug-related AEs, or there is one subject experiencing drug-related SAE.

5. Definition of end of study

If the subject has completed all stages of the study, including the last visit, it is considered that the study has been completed. The study completion is defined as the date of the last visit for the last subject during the study period.

Study flow chart

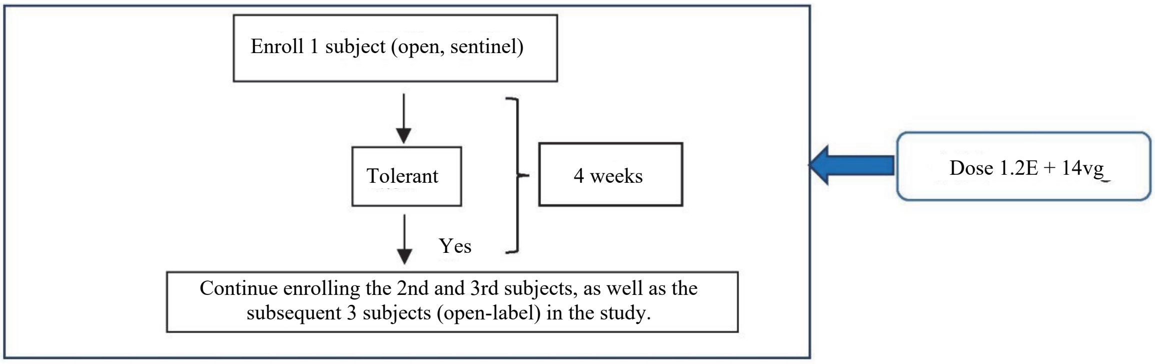

Fig. 4 Study flow chart

2. Sample size and study design

The recruitment plan for this study is to enroll 10 subjects.

3. Study duration

The duration of the continuous observation period for this study is 52 weeks.

4. Subject selection

1) Inclusion criteria

Subjects must meet all of the following criteria to be eligible for enrollment in this study.

(1) During the screening process, subjects must be between the ages of 6 months and 18 years (excluding 18 years old), with no gender restrictions;

(2) Clinically diagnosed with type 2 or type 3 5q SMA;

(3) Type 2 patients lack the ability to walk, while type 3 patients either lack the ability to walk or possess the ability to walk but are unable to squat and stand up independently;

(4) Possessing the ability to sit independently (defined as: sitting unaided for more than 5 seconds with the support of one's own upper limbs);

(5) One or both hands can only be raised to the height of the mouth;

(6) Negative for SMN2 gene modification mutation (c.859 G>C);

(7) The subjects or their guardians are able to understand and are willing to comply with the study protocol requirements and procedures, voluntarily participate, and sign the informed consent form.

2) Exclusion criteria

The presence of any of the following will render the subject ineligible for this study.

(1) Having participated in any other gene therapy clinical studies;

(2) Unable to participate in the late-stage follow-up of the study;

(3) Within 30 days prior to the screening period, the use of SMN2-targeted modulating agents (Spinraza

or Risdiplam) has occurred;

(4) Serum anti-AAV9 neutralizing antibody titer > 1:10 (if serum anti-AAV9 neutralizing antibody titer > 1:10, then cerebrospinal fluid anti-AAV9 neutralizing antibody titer will be measured. If cerebrospinal fluid anti-AAV9 neutralizing antibody titer >1:10, the subject will be ineligible for this study);

(5) Patients with a predisposition to allergies, including those with hypersensitivity or allergic reactions to prednisolone or other corticosteroids, or their excipients, as well as those with hypersensitivity or allergic reactions to iodine or iodine-containing products;

(6) Presence of contraindications for spinal puncture procedure or intrathecal treatment;

(7) X-ray examination reveals severe scoliosis of the spine (≥ 50°);

(8) Planning or anticipating to undergo scoliosis correction surgery within 1 year after administration of the medication;

(9) Invasive ventilation support or blood oxygen saturation < 95%;

(10) Severe non-respiratory diseases requiring systemic treatment and/or hospitalization within two weeks prior to the initiation of the study;

(11) Occurrence of respiratory infections requiring medical care, medical intervention, or any increase in supportive care in any manner within four weeks prior to the study;

(12) Occurrence of severe non-pulmonary/respiratory infections or concomitant diseases within four weeks prior to the administration of the investigational product, which, in the opinion of the principal investigator (PI), may pose unnecessary risks to gene transfer, such as significant renal or liver impairment, known epilepsy, diabetes, idiopathic hypocalciuria, or systemic cardiomyopathy;

(13) History of bacterial meningitis or brain or spinal cord diseases, including tumors, or magnetic resonance imaging (MRI) or computed tomography (CT) abnormalities that may interfere with lumbar puncture (LP) or cerebrospinal fluid circulation;

(14) Unable to discontinue the use of laxatives or diuretics within 24 hours prior to drug administration;

(15) Any other serious or active diseases besides SMA, which the investigator believes may interfere with the subject's treatment, evaluation, or compliance with the study protocol. Examples include cardiovascular diseases, kidney diseases, liver diseases, endocrine disorders, malignant tumors, diabetes, hypersensitivity, infectious diseases, coagulation abnormalities, immunodeficiency disorders, and severe mental and neurological disorders;

(16) Positive for human immunodeficiency virus (HIV) antibodies, hepatitis B surface antigen, hepatitis C antibodies, or Treponema pallidum antibodies;

(17) History of alcohol consumption and drug abuse;

(18) The investigator considers the subject unsuitable for inclusion in this study.

3) Withdrawal of subjects and relevant management

(1) **Withdrawal at investigator's discretion**

The withdrawal of a subject from the study refers to the situation where an enrolled subject encounters circumstances that make it inappropriate to continue the study, and the investigator decides that the subject should withdraw from the study.

- The investigator deems it necessary to terminate the study from a medical ethics perspective;
- If a SAE occurs, the subject should not continue in the study;
- The investigator determines that withdrawal from the study is in the best interest of the subject.
- Poor subject compliance, including the following situations:

a. The study subject does not adhere to the prescribed medication regimen and undergoes examinations as required;

b. The subject uses other drugs or food that affect safety evaluation and PK (viral load) test results;

c. The subject has smoking and drinking behaviors (the investigator will determine whether withdrawal is necessary);

d. The subject has other behaviors that may affect the study outcomes.

(2) **Withdrawal by subject**

In accordance with the provisions of the informed consent form, the subject has the right to withdraw from the study at any time, or the subject may be considered withdrawn (also referred to as dropout) if they do not revoke their informed consent but no longer accept medication and testing, resulting in loss to follow-up. The investigator should make every effort to understand the reasons for withdrawal and document them accordingly.

(3) **Management for withdrawal of subjects**

Subjects who withdraw from the study during the screening period prior to randomization will be considered screening failures. In the source documents, they must be recorded as screening failures and no further follow-up is required.

Subjects who terminate or withdraw from the study after randomization will be considered as "early withdrawal from the study", and the primary reasons for withdrawal will be recorded as one of the following: AEs, loss to follow-up, protocol violation, death, or other. Investigators must make appropriate efforts to contact lost-to-follow-up subjects, with at least three phone calls. If a subject using the study drug withdraws early from the study, the following assessments will be performed: vital signs, physical examination, 12-lead electrocardiogram, hematology, blood chemistry, and urinalysis. In addition, for subjects who have been randomized but withdraw from the study for any reason before receiving the study drug, no "early withdrawal from the study" assessments are required for the subject.

IV. Study methods

1. Investigational product information

Drug name: GC101

Dosage form: Injection

Appearance: Colorless transparent to opalescent liquid

Strength: 1 mL/vial

Titer: 1.2×10^{14}vg/mL

Storage condition: ≤ -70 °C

Batch No.: According to the actual batch

2. Packaging and labeling of investigational product

Unified format will be used for investigational product labeling, and the content of the secondary packaging label includes:

- Investigational product name ("For Clinical Study Use Only" should be marked)
- Protocol number
- Drug number
- Strength
- Batch No.
- Expiry date
- Storage
- Sponsor, etc.

Table 3. Label on the secondary packaging box of investigational product

Drug name:	GC101 (for clinical study use only)		
Protocol number:	JLJY-GC101-SMA-005	Drug number:	GC101
Strength:	1 mL: 1.2×10^{14} vg	Expiry date:	24 months
Batch No.:	According to actual batch	Storage:	≤ -70 ℃
Sponsor:	Beijing GeneCradle Technology Co., Ltd.		
Note:	If you have any questions, please contact the investigator promptly. Unused medications must be collected and returned in a unified manner.		

Table 4. Label of investigational product vials

Drug name:	GC101 (for clinical study use only)
Batch No.:	According to actual batch
Strength:	1 mL, 1.2×10^{14} vg
Sponsor:	Beijing GeneCradle Technology Co., Ltd.
Manufacturer:	Beijing GeneCradle Technology Co., Ltd.

3. Investigational product administration

1) Prior to injection, warm the drug to room temperature and quickly place it in a 37 °C water bath to thaw. After 5 minutes, the drug should be melted and appear as a colorless, transparent to opalescent liquid. If any particulate precipitation is observed, do not use the drug.

2) After thawing, the drug must be used within 1 hour. If delayed use is required, store it at 2-8 °C and use it within 7 days.

3) Adjust the injection volume to 2 mL using sterile physiological saline for injection, following aseptic

procedures.

4) Intrathecal injection.

5) After completing the injection, press the injection site for 5 minutes. If there is still fluid leakage, continue to apply pressure.

6) Subjects should rest in supine position.

4. Receipt, dispensing, and storage of the investigational product

The investigational product is provided free of charge by Beijing GeneCradle Technology Co., Ltd. (hereinafter referred to as GeneCradle) and distributed to clinical centers according to the plan. After the investigator completes the recruitment of subjects and confirms their enrollment, the person in charge of the Clinical Medicine of GeneCradle must be informed. GeneCradle will send the investigational product within 48 hours after receiving the notification (may be postponed on weekends). The drug shipment is entrusted to a third-party logistics company, and the drug will be transported with dry ice insulation, and monitored for temperature throughout the entire process. The drug recipient must carry out the corresponding acceptance work according to the logistics requirements.

Table 5. Drug delivery/receiving institution information

**Institution name**	**Contact**	**Tel.**
The Seventh Medical Center of the Chinese People's Liberation Army General Hospital	Yu-Min Yang	66721296
Beijing GeneCradle Technology Co., Ltd.	Zhi-Ming Zhu	17600679965

The investigational product must be stored by the clinical study site's drug administrator according to the required storage conditions. Clinical study personnel must ensure that the drug is only used for the specific clinical study. The investigational product is to be distributed and recorded on the clinical study drug dispensing list based on prescriptions or requisition forms issued by physicians participating in the study and obtained from the drug administrator. The investigational product should be administered to the study subjects according to the protocol, and the aluminum cap and rubber stopper must not be removed when drawing the drug solution. After administration, the subject's identification number should be marked on the bottle for future traceability. Any remaining drug, along with its packaging, should be promptly returned to the drug administrator and recorded on the remaining drug inventory list. The remaining drug should be stored long-term at a temperature of ≤ -70 °C.

At the end of the clinical study, the drug administrator should collect the outer packaging of the investigational product (including the packaging box and empty bottles) and any remaining study drug. The quantity of the collected drug should be recorded, and the remaining drug and outer packaging should be returned to the sponsor. The transportation process should be entrusted to a third-party logistics provider, using dry ice for temperature control during transit, and accompanied by a handover list.

Gene basket investigator contact: see Table 5.

V. Concomitant medications

In the study protocol, prophylactic oral prednisolone will be administered at a dose of 1 mg/kg body weight, with a maximum dose not exceeding 60 mg/day (starting one day before the intrathecal injection). The medication will be taken daily and continuously for at least 4 weeks. The changes in AST and ALT levels in the blood will be monitored, and if no significant fluctuations are observed, the dose will be gradually reduced and eventually discontinued. If a statistically significant elevation in enzyme activity (> 2×ULN) occurs, the medication should be continued until the values return to normal range and remain stable for at least one week, after which the dose will be gradually reduced and discontinued.

1. Permitted medications

Investigators may use their discretion to determine whether over-the-counter (OTC) medications can be used to treat self-limiting conditions (e.g., using Tylenol for fever) following administration of the investigational product. Approval from the investigator must be obtained before using any OTC medications in combination. During the entire study treatment period, all prescription medications must be approved by the investigator. The importance of consulting the investigator before using any medications during the study should be communicated to all subjects. Caution should be exercised when using concomitant medications with a narrow therapeutic window, such as digoxin, and adverse reactions should be monitored.

All medications used by the subjects during the study period must be documented in the subject's Case Report Form (CRF).

2. Prohibited medications and treatments

It is prohibited to use any investigational drugs within 28 days prior to Day 0 (baseline). During the study, if the subject uses the oral solution of nusinersen and risdiplam, or undergoes liver or any other organ transplantation, the subject will be withdrawn from the study.

3. Rescue medication

The investigator, at their discretion, must determine the use of medication for AE and provide standard medical support treatment to manage the AE.

VI. Observation measures and time of examination

1. Demographic information and medical history

During the screening process, the investigator should thoroughly understand the subject's medical history, as well as their smoking, drinking, and past medication history. The collection of demographic information for the subject includes gender, age, height, weight, and other demographic data. Particular attention should be paid to the subject's past medical history and the results of their most recent physical examination.

2. Safety measures

(1) Adverse events

The evaluation of AE involves the type, incidence, severity, onset and end dates, whether it is a SAE, the relationship to the investigational product, and the outcome.

(2) Vital signs

Vital signs: body temperature, blood pressure, pulse, and respiration.

(3) Physical examination signs

A comprehensive physical examination will be conducted at the screening and protocol-specified time points, which should include at least the following assessments: general appearance, skin, head and neck (including thyroid), eyes, ears, nose, throat, chest, abdomen, lymph nodes, and neurological system examination.

(4) Electrocardiogram

A standard 12-lead electrocardiogram (ECG) examination should be performed. Any clinically significant ECG

abnormalities detected after enrollment should be recorded as AEs.

(5) Echocardiography

Perform a standard pediatric transthoracic color echocardiography examination, including cardiac structure, blood flow, left ventricular systolic function, etc. Any clinically significant echocardiographic abnormalities found after enrollment should be recorded as AEs.

(6) Viral shedding (viral load)

Collect blood for viral shedding detection, where the blood sample used for PK will be the same, without additional collection.

(7) Laboratory tests

A. Infection screening

During the screening period, tests for HBsAg, HIV antibodies, HCV antibodies, and syphilis treponemal antibodies will be conducted.

B. Hematology, blood chemistry, urinalysis, and coagulation function tests

During the screening and study periods, laboratory tests such as hematology, coagulation function, blood chemistry, and urinalysis will be conducted according to the protocol. Specific test items are listed in the table below.

Hematology + coagulation	Blood chemistry	Urinalysis	Other
Hemoglobin<br>Hematocrit<br>RBC count<br>MCV<br>MCH<br>MCHC<br>WBC count<br>Platelet count<br>Total neutrophil count (Abs)<br>Eosinophilic Granulocytes (Abs)<br>Monocytes (Abs)<br>Basophil Count (Abs)<br>Lymphocytes (Abs)<br>Coagulation Function (PT, APTT, INR, ...)	BUN and creatinine<br>Glucose (fasting)<br>Na, Ka, Ca, Cl<br>Total CO_2<br>AST, ALT<br>Total bilirubin<br>Alkaline phosphatase<br>Lactate dehydrogenase<br>Alpha-hydroxybutyratase<br>Creatine kinase<br>Creatine kinase isoenzyme<br>Gamma-glutamyl transferase (GGT)<br>Uric acid<br>Urea<br>Albumin<br>Total protein<br>Total cholesterol<br>Troponin I<br>Myoglobin	Protein<br>Nitrite<br>White blood cells (qualitative)<br>Occult blood<br>Microscopic examination and culture (only when urine dipstick analysis is positive)	AAV9 neutralizing antibody titer<br>AVV9 binding antibody titer<br>SMN binding antibody titer<br>Cytokines (IL-2, IL-4, IL-6, IL-10, IFN-γ, TNF-α)<br>PK

- Chest x-ray

A chest x-ray will be performed at screening.

2. Efficacy measures

(1) Hammersmith Functional Motor Scale Expanded (HFMSE) for neuromuscular function assessment

HFMSE will be conducted at the following time points: pre-administration (screening phase), post-administration on Day 28 (4 weeks), Day 56 (8 weeks), Day 84 (12 weeks), Day 112 (16 weeks), Day 196 (28 weeks), and Day 364 (52 weeks).

(2) Revised Upper Limb Module (RULM) for neuromuscular function assessment

Neuromuscular function assessments (RULM) will be conducted at the following time points: pre-administration (screening period), post-administration on Day 28 (4 weeks), Day 56 (8 weeks), Day 84 (12 weeks), Day 112 (16 weeks), Day 196 (28 weeks), and Day 364 (52 weeks).

(3) Pulmonary function;

Lung function assessment will be performed at screening period and at the end of study (52 weeks).

(4) MRI muscle examination;

During the screening period and the study period (52 weeks), MRI muscle examinations will be conducted.

(5) Electromyography

Electromyography examinations will be conducted at the following time points: pre-administration (screening phase), Day 7 (1 week) post-administration, Day 28 (4 weeks), Day 56 (8 weeks), Day 112 (16 weeks), Day 196 (28 weeks), and Day 364 (52 weeks).

3. Pharmacokinetic (viral load) endpoints

Utilizing a validated quantitative PCR method, the viral titers of GC101 in serum will be detected, and based on the PK data from the previous dose group, adjustments can be made to the blood sampling time points for the subsequent dose group. The sample processing procedure will be carried out according to the sample operation manual provided by the relevant testing unit. PK parameters include $AUC_{0\text{-inf}}$, $AUC_{0\text{-t}}$, C_{max}, T_{max}, kel, $t_{½}$, CLz, Vz, and AUC_%Extrap, which are derived from the blood drug concentration-time data.

4. Immunogenicity measures

ADA testing, measurement of anti-AAV9 neutralizing antibodies and SMN antibodies in serum, as well as measurement of cytokines of IL-2, IL-4, IL-6, IL-10, IFN-γ, and TNF-α in serum will be conducted.

VII. Follow-up time

1. The study period from week 1 to week 4 is designated as the medication observation phase.

2. Week 5-week 52 is the follow-up period

During the study period, a total of 6 outpatient follow-up visits will be scheduled, including: week 6 (±3 days), week 8 (±6 days), week 12 (±9 days), week 16 (±9 days), week 28 (±15 days), and week 52 (±30 days).

3. Week 53 to 5 years is the long-term follow-up period.

After the completion of the 52-week study (week 52), the subjects will choose for voluntary participation in the long-term follow-up study.

VIII. Clinical evaluation

Safety evaluation

Vital signs (D1-7, D14, D28, D42, D56, D84, D112, D196, D364).

Physical examination (post-administration Day 7, Day 14, Day 28, Day 42, Day 56, Day 84, Day 112, Day 196, Day 364).

Hematology (CBC), blood chemistry, coagulation, and urinalysis (on Day 7, Day 14, Day 28, Day 42, Day 56, Day 84, Day 112, Day 196, and Day 364 post-administration).

12-lead electrocardiogram (post-administration on Day 7, Day 14, Day 28, Day 42, Day 56, Day 84, Day 112, Day 196, Day 364).

Echocardiogram (pre-administration (screening phase), post-administration D7, D28, D84, D364).

PK blood sampling (Day 7 post-administration, Day 14±3 days, Day 28±3 days, Day 42±6 days, Day 56±6 days, Day 84±9 days, Day 112±10 days, Day 364±30 days).

Immunogenicity sampling involves the detection of anti-AAV9 neutralizing antibodies and SMN antibodies in serum; cytokine detection, and measurement of serum levels of IL-2, IL-4, IL-6, IL-10, IFN-γ, and TNF-α (at Day 14±3, Day 56±6, and Day 112±10 post-administration).

Efficacy evaluation

Neuromuscular assessment using the HFMSE method [before administration (screening period), after administration on Day 28 (4 weeks), Day 56 (8 weeks), Day 84 (12 weeks), Day 112 (16 weeks), Day 196 (28 weeks), and Day 364 (52 weeks)].

Neuromuscular assessment using the RULM method [pre-administration (screening phase), post-administration D28 (4 weeks), D56 (8 weeks), D84 (12 weeks), D112 (16 weeks), D196 (28 weeks), D364 (52 weeks)].

Electromyography (post-administration on Day 7, Day 28, Day 56, Day 112, Day 196, and Day 364).

Note: The neuromuscular assessment must be independently examined and recorded by two investigators on the

same day.

IX. Known and potential risks and benefits of the project, and risk management plan

1. Benefit/risk assessment

(1) Benefit

The benefits of GC101 include:

① The non-clinical study of the GC101 treatment group demonstrated significant efficacy and potential for long-term benefits. Theoretically, GC101 can improve the clinical symptoms of SMA and alter the natural disease progression.

(2) Risk

This drug has not yet undergone clinical studies, but internationally, there are similar clinical studies on drugs and non-clinical toxicology study results of GC101 that can be used to assess potential risks for this study. The identified treatment-related risks of GC101 or safety issues considered as potential risks are discussed below:

A. Elevated liver enzymes and hepatic failure

Since the active ingredient in the GC101 formulation is a recombinant adeno-associated virus vector, there is a T-cell immune response to the recombinant viral vector in humans, which may lead to transient elevations in liver enzymes (transient hepatitis). In patients treated with Zolgensma, there have been two cases of death due to liver failure. However, international clinical experience has shown that this transient hepatitis can be effectively managed with corticosteroid medications (such as prednisone), with a favorable prognosis. Liver failure is a more severe complication, carrying a risk of death.

B. Transient fever

Due to the active ingredient in the GC101 formulation being a recombinant protein product (viral vector), there is a possibility of hypersensitivity reactions or transient fever in the body. This is a normal phenomenon for this class of drug products and can be managed through close monitoring and intervention for hypersensitivity reactions. The prognosis is favorable.

C. Thrombotic microangiopathy

Following the market launch of the similar drug Zolgensma, data showed that among 800 patients treated with Zolgensma, a total of 5 cases of thrombotic microangiopathy were reported to have occurred within 6-11 days of drug infusion. The clinical manifestations included vomiting, hypertension, oliguria/anuria/edema, and laboratory data showed a decrease in platelets, elevated serum creatinine, proteinuria/hematuria/hemolytic anemia. One patient died 6 weeks after the event.

In the single-dose toxicity study of GC101 adeno-associated virus injection in C57BL/6N mice and cynomolgus macaques, no related AEs were observed. GC101 adeno-associated virus injection has not yet been tested in clinical studies, and there is currently no clinical data on related AEs. Investigators will closely monitor the clinical symptoms, signs, urinalysis, platelet count, and coagulation profile + D-dimer in pediatric patients within 1-2 weeks of treatment. If thrombocytopenia, coagulation disorder, and elevated D-dimer levels occur, further evaluation will be conducted, including diagnostic testing for hemolytic anemia and renal insufficiency.

D. Immunogenicity

GC101 adeno-associated virus injection is a biological product, essentially a protein, with immunogenic properties. When the biological product enters the human body, the immune system recognizes it as an antigen, triggering an immune response. Ultimately, B lymphocytes produce corresponding drug-resistant antibodies.

In the single intrathecal injection study of GC101 adeno-associated virus injection in cynomolgus macaques, an increase in AAV9 and SMN antibody titers was observed after the administration of the investigational product, but no associated toxic reactions were found.

During the screening phase, serum anti-AAV9 neutralizing antibody titers will be tested. If the titer is > 1:10, the subject will be excluded. Concurrently, investigators will continuously observe the therapeutic effect of the investigational product on SMA patients throughout the study.

E. Cardiotoxicity

The mechanism leading to cardiac toxicity is not yet clear. It may be due to the presence of T-cell immune responses in the human body against the recombinant viral vector, resulting in the overactivation of T-cells and subsequently causing cardiac toxicity.

In a single-dose toxicity study of GC101 adeno-associated virus injection in C57BL/6N mice and cynomolgus macaque models, no related AEs were observed. In a non-clinical toxicity study of a similar drug, Zolgensma, in mice, reported findings included cardiac degeneration, fibrosis, and atrial thrombus formation.

Before administering the medication, the investigator will screen the pediatric patients for any history of heart disease. After the medication is given, the investigator will monitor heart-related symptoms/parameters, including heart rate, blood pressure, cardiac biomarkers, electrocardiogram, and echocardiogram.

F Thrombocytopenia

The mechanism leading to thrombocytopenia is not yet clear. This product is an adeno-associated virus-mediated SMN1 gene replacement therapy drug. It is possible that the adeno-associated virus activates platelets and induces the formation of platelet-leukocyte aggregates. Furthermore, the adeno-associated virus can activate endothelial cells, producing procoagulant stimulation, leading to thrombocytopenia and platelet dysfunction.

In a single-dose toxicity study of GC101 adeno-associated virus injection in C57BL/6N mice and cynomolgus macaques, no related AEs were observed. In a Phase III clinical study of a similar drug, Zolgensma, a decrease in the mean platelet count relative to baseline was observed at multiple time points in the study subjects, but no clinically significant events were noted. In a Phase III clinical study of the similar drug Zolgensma, one out of 33 subjects experienced thrombocytopenia.

Investigators will closely monitor the pediatric patients' platelet count before and after medication administration. If any abnormalities in platelet count are detected, appropriate therapeutic measures will be taken promptly.

G. Dorsal root ganglion cell inflammation

The mechanism of adeno-associated virus-induced dorsal root ganglion cell inflammation is not yet clear. This product is an adeno-associated virus vector-mediated SMN1 gene replacement therapy drug. The inflammation of dorsal root ganglion cells may be related to the adeno-associated virus vector. Dorsal root ganglion neurons have a response to acute inflammatory factors, and adeno-associated virus infection can enhance the expression of

inflammatory mediators such as TNF-α and IL-1, leading to dorsal root ganglion cell inflammation.

In the toxicity study of GC101 adeno-associated virus injection using C57BL/6N mice and cynomolgus macaques (macaca fascicularis) as models, no related AEs were observed. In the single intrathecal injection toxicity study of Zolgensma conducted by Novartis in cynomolgus macaques, it was found that at a dose of 3.0×10^{13} vg/animal, most subjects exhibited mild to moderate inflammatory cell infiltration in the dorsal root ganglia. In multiple subjects, neuronal necrosis, degeneration, and disappearance were observed, ultimately confirming the pathological changes were associated with the intrathecal injection of Zolgensma. No related AEs were found in the clinical study data of Zolgensma.

Investigators will closely monitor pediatric patients before and after medication administration for any local pain, sensory disturbances, or worsening motor impairments. If dorsal root ganglion inflammation occurs, anti-inflammatory and neurotrophic treatments will be administered as appropriate. Cerebrospinal fluid examination may be performed if necessary, based on clinical conditions.

H. Potential risks or discomforts associated with the study procedures

Blood draws, intrathecal injections, electromyography, electrocardiography, echocardiography, magnetic resonance imaging, etc., may cause fear, crying, breath-holding episodes, or respiratory suppression in children. Investigators will strictly follow the operational guidelines, prepare in advance, develop contingency plans, and arrange for experienced medical professionals to be responsible for the procedures, ensuring the safety of the children and minimizing their pain.

I. Unknown risks

The pediatric patients may experience adverse reactions or discomforts not listed in this document. Some adverse reactions may not have been discovered yet, and there may be unforeseen adverse reactions in the short or long term, even life-threatening serious events. The investigators will closely monitor the patients, provide appropriate care based on the situation, implement emergency measures, and transfer the patients to the intensive care unit for treatment if life-threatening situations arise.

J. Treatment ineffectiveness

GC101 adeno-associated virus injection has been proven safe and effective in rodent and non-human primate cynomolgus macaque animal studies. However, clinical studies for this project have not yet been conducted, and there is a possibility of treatment ineffectiveness.

2. Adverse events

(1) Definition of adverse events

AE: refers to an unfavorable medical event that occurs in a subject after receiving a drug, but not necessarily having a causal relationship with the treatment.

This study commences with the signing of the informed consent form by the subjects and includes the documentation of any adverse medical events occurring during the long-term follow-up period, regardless of whether there is a causal relationship with the study drug. This documentation should cover symptoms, signs, diagnoses, and abnormal laboratory tests, among other aspects.

Investigators should meticulously document any AEs experienced by the study subjects and the corresponding

actions taken. The documentation of AEs should include: a description of the AE and all related symptoms, the time of occurrence, the severity, the seriousness, the duration, the measures taken, the final outcome, and the relationship to the study drug.

(2) Observation, recording, and reporting of adverse events

Observation and documentation: Investigators should carefully observe and document any AEs occurring during the clinical study in subjects. They should ask subjects to truthfully report their actual experiences after taking the medication, avoiding leading questions. Attention should be paid to observing adverse reactions or unanticipated toxic side effects (including symptoms, signs, and laboratory tests). AEs should be documented in detail in the case report form, regardless of whether they are related to the study drug. This includes the name of the AE, the time of occurrence, severity, duration, laboratory test measures, management methods, treatment course, outcomes, follow-up times, etc. Additionally, the concomitant medication usage should be documented in detail to facilitate the analysis of the relationship between AEs and the study drug. The documentation should be signed and dated.

For the terminology for AE names, CTCAE version 5.0 or higher will be referred to, with preference given to recording diagnostic names. If a clear diagnosis cannot be determined, symptoms/signs should be recorded instead. Pre-existing medical conditions or diseases that worsen or become aggravated after the initiation of the study should be managed as AEs.

In this study, significant abnormalities in clinical laboratory tests, vital signs, and ECGs, as determined by the investigator, are reported as AEs with clinical significance.

Abnormal laboratory test values should first be compared by the investigator to the baseline values and assessed for clinical significance. Abnormal laboratory test values that are deemed clinically significant and show a worsening or aggravating trend compared to the baseline should be reported as AEs, and followed up until they return to normal or baseline levels. Abnormal laboratory test values that are not clinically significant or are clinically significant but show no change compared to the baseline period should not be reported as AEs.

Medical management of study subjects: When an AE is detected, the investigator may take necessary measures according to the subject's condition, such as adjusting the dosage or temporarily suspending medication, and decide whether to terminate the study. In the event of a SAE, the institution responsible for the study must immediately take necessary measures to protect the safety of the subjects.

During the clinical study follow-up period, all AEs should be followed up until recovery, return to baseline, stabilization of the condition, a reasonable explanation is obtained, or the subject is lost to follow-up as the end time of the AE. If the AE still exists at the last follow-up and is classified as very likely or possibly related to the study drug, or cannot be evaluated, it is necessary to track until recovery, return to baseline, stabilization of the condition, a reasonable explanation is obtained, or the subject is lost to follow-up.

(3) Judging the severity of adverse events

The severity of AEs will be determined according to the NCI Common Terminology Criteria for Adverse Events (CTCAE V5.0 or higher).

CTCAE Version 5.0: severity grading of adverse events in clinical studies:

Grade 1: mild; asymptomatic or mild symptoms; clinical or diagnostic observations only; no treatment required.

Grade 2: moderate; requiring minimal, localized, or non-invasive treatment; limitations in age-appropriate instrumental activities of daily living[a].

Grade 3: severe or medically significant but not immediately life-threatening; resulting in hospitalization or prolongation of hospitalization; causing disability; limiting self-care activities of daily living[b].

Grade 4: life-threatening; requires urgent treatment.

Grade 5: deaths related to AEs.

Note: Activities of daily living (ADL): [a]instrumental activities of daily living refer to activities such as cooking, purchasing clothing, using the telephone, and managing finances; [b]self-care activities of daily living refer to activities such as bathing, dressing, eating, grooming, and taking medication, without being bedridden.

(4) Criteria for judging the relationship between adverse events and study drug

AEs and their relationship to the study drug can be determined according to the following five-level classification criteria:

Definitely related: The reaction appears in a reasonable time sequence after drug administration, and the reaction is consistent with the known reaction types of the suspected drug. The condition improves after discontinuation of the drug (the reaction reappears upon re-administration). The reaction cannot be explained by the subject's disease or concomitant medication.

Probably related: The reaction appears in a reasonable time sequence after drug administration, and the reaction is consistent with the known reaction types of the suspected drug; improvement occurs after discontinuation of the drug, and it cannot be explained by the investigator's disease or concomitant medication.

Possibly related: The reaction occurs in a reasonable time sequence following drug administration, but the subject's clinical condition or other therapeutic interventions may also contribute to the occurrence of the reaction.

Possibly unrelated: The occurrence of the reaction does not quite correspond to the reasonable time sequence after drug administration, and the reaction does not quite match the known reaction types of the suspected drug; the clinical status of the investigator or other treatment methods may potentially cause this reaction.

Definitely unrelated: The reaction occurs in an unreasonable time sequence after drug administration and does not correspond to the known reaction types of the suspected drug. The subject's clinical condition or other treatment methods may cause this reaction. The reaction disappears when the disease condition improves or other treatments are stopped, and reappears when other treatments are used again.

(5) Management of adverse events

When AEs occur in study subjects, acceptable clinical treatment measures should be employed. All AEs should be followed up until recovery, return to baseline, stabilization of the condition, a reasonable explanation is obtained, or the subject is lost to follow-up.

3. Serious adverse events

(1) Definition of serious adverse events

SAE refer to AEs that lead to the following outcomes:

A. Leading to death

B. Life threatening

C. Leading to hospitalization or prolongation of hospital stay

D. Resulting in permanent or significant disability or functional impairment for the subject

E. Leading to congenital malformations or birth defects

F. Other medically significant events

Important medical events are those that may not immediately endanger life, result in death, or require hospitalization, but are generally considered serious if medical intervention is needed to prevent one of the aforementioned situations from occurring. Examples include significant treatment in the emergency room, allergic bronchospasm occurring at home, non-hospitalized cases of malaise or seizures, and the development of drug dependence or addiction.

In the event of a SAE occurring after the completion of the long-term follow-up in the clinical study and before the approval conclusion of this project is obtained, the investigator should report to the sponsor. If it is an unexpected serious adverse reaction, the sponsor should promptly report it to the regulatory authorities.

(2) Management of serious adverse events

Upon the occurrence of an SAE, the investigator should promptly take necessary measures to ensure the safety and rights of the study subjects. Subjects should be closely monitored until recovery, return to baseline, stabilization, or loss to follow-up, and the relevant information should be recorded in the Serious Adverse Event Report Form.

(3) Recording, reporting, and follow-up of serious adverse events

After the stabilization of the study subject, the investigator should promptly record the SAE-related information in the Serious Adverse Event Report Form, including the description of the clinical course of the SAE, assessment of severity, seriousness, causality with the study drug or study procedure, measures taken, and the date of event resolution. Investigators must provide their assessment of causality when reporting SAEs and promptly report to the drug regulatory authorities. If the investigator cannot determine whether an AE is an SAE, it should be considered an SAE until its nature can be proven otherwise. Reporting of SAEs should be conducted according to the time limits and requirements for reporting.

Table 6. Contact information for reporting serious adverse events

Beijing GeneCradle Technology Co., Ltd.	Contact Person: Zhu Zhiming<br>Tel: 17600679965<br>E-mail: zhuzm@bj-genecradle.com
The Seventh Medical Center of the Chinese People's Liberation Army General Hospital	Contact Person: Ma Xiuwei<br>Tel: 13651111080<br>E-mail: pony007@vip.sina.com
Medical Administration and Management Bureau, National Health Commission of the People's Republic of China,	Tel: 010-668792201<br>Fax: 010-68792734<br>E-mail: saefax@163.com
Drug Study Supervision Office, Drug Registration Division, National Medical Products Administration	Tel: 010-88331134<br>Fax: 010-88363228<br>E-mail: yjjdc@nmpa.gov.cn
Drug Registration Office, Beijing Municipal Medical Products Administration	Tel: 010-83979465<br>Fax: 010-83560723

4. Unexpected serious adverse reactions (SUSAR)

In accordance with the requirements for rapid reporting of "unexpected serious adverse reactions" specified in the *Standards and Procedures for Rapid Reporting of Safety Data during Drug Clinical Studies*, unexpected serious adverse reactions in this study protocol refer to suspected and serious adverse reactions, of which the nature, severity, etc., exceed the range of adverse reaction information already included in the Investigator's Brochure for the investigational product.

X. Quality control and quality assurance in the study

Establish a comprehensive quality assurance system, clearly defining the responsibilities of all parties involved, such as the investigator institutions and the sponsors.

During the design and implementation stages of the study, necessary steps should be taken to ensure the accuracy, authenticity, and completeness of the collected data. All observed data in the clinical study should be promptly recorded and verified to ensure data reliability.

The investigator should conduct a systematic inspection of clinical study-related activities and documents to evaluate whether the study is conducted in accordance with the study protocol, SOPs, and relevant regulatory requirements, and whether the study data is recorded in a timely, truthful, accurate, and complete manner.

The sponsor or sponsor-authorized investigator may conduct systematic inspections of the clinical study. Inspections will be carried out by personnel not directly involved in the project, and the privacy and data of the subjects will be strictly protected.

11. Data storage

All study materials for this study should be archived and preserved in a timely manner. Investigators/clinical study institutions and sponsors should archive and preserve the materials in accordance with current GCP, the "Guidance on Essential Documents for Drug Clinical Studies" (No. 37, 2020), and relevant SOPs, for at least 5 years after the investigational product has been approved for marketing. Without the written consent of the sponsor, investigators/clinical study institutions shall not provide the materials to any third party outside of the collaborating institutions in any form.

XII. Data safety monitoring

1. Data management

(1) Case report form (CRF): design the CRF according to the study protocol.

(2) Data collection: investigators collect subject visit data according to the requirements of the study protocol and record it accurately, promptly, completely, and in a standardized manner.

(3) Data entry: CRF data is derived from the original records.

(4) Source data verification (SDV): the investigator conducts a consistency check between the CRF data and the source data. Any issues identified can be raised as queries.

2. External data transfer

Sign the external data transfer agreement and conduct external data management.

3. Medical coding

In this study, AEs are coded using the MedDRA dictionary, and concomitant medications are classified as anatomical according to the ATC coding principles.

XIII. Statistical processing

Data input and modification, statistical analysis.

1. General principles

Quantitative data are generally described using mean, median, standard deviation, maximum, and minimum values, while count data or ordinal data are described using frequency and frequency distribution.

2. Analysis dataset

Safety set (SS): all enrolled subjects who have received the investigational drug and have safety outcome records.

Full analysis set (FAS): all enrolled subjects who have received the investigational drug in the study.

Pharmacokinetics concentration set (PKCS): all enrolled subjects who have received the investigational drug and have at least one valid post-dose blood drug concentration data during the study period.

Pharmacokinetics parameter set (PKPS): all enrolled subjects who have received the investigational drug and have at least one valid PK parameter during the study period. The exclusions from the PKPS also include a) those with serious protocol violations affecting the PK parameter results or those with unmeasurable parameters; b) subjects with pre-dose concentrations greater than 5% of C_{max}.

Pharmacodynamics observation set (PDOS): all enrolled subjects who have received the investigational product and have at least one valid PD outcome during the study period.

3. Safety analysis

AEs will be coded using MedDRA and will be classified and summarized at two levels: System Organ Classification (SOC) and Preferred Term (PT).

This study primarily conducts statistical analysis on TEAEs occurring after drug administration. AEs occurring before drug administration will be listed in the form of a checklist.

The occurrence frequency, number of cases, and incidence rate of all AEs, drug-related AEs, SAEs, and AEs leading to discontinuation separately after administration will be calculated.

The severity of AEs and their relationship to the study drug will be listed. The AEs related to the study drug, AEs

unrelated to the study drug, AEs leading to discontinuation, and SAEs will be listed, respectively.

A statistical description of the changes in vital sign indicators from baseline to each time point after treatment will be provided, respectively, including the number of subjects, mean, standard deviation, median, minimum, and maximum values.

In the form of a cross-table before and after administration, the changes in laboratory tests, physical examinations, and 12-lead electrocardiograms from baseline to each time point after administration will be listed. Abnormal laboratory tests, physical examinations, and 12-lead electrocardiogram examinations at each time point after administration will be listed in a tabular format.

4. Efficacy analysis

The changes in neuromuscular function scores (HFMSE) and neuromuscular function scores (RULM) at each evaluation time point for subjects compared to baseline will be calculated.

5. Pharmacokinetic (viral load) analysis

Key PK data point agreements: a) sampling time points will be calculated based on the actual sampling time; b) all blood drug concentration values below the lower limit of quantification (LLOQ) are treated as "0" for PK analysis before T_{max}, and as missing after T_{max}. However, during descriptive statistical analysis, they are treated as 0, and the number of BQL (below the limit of quantification) at each time point should be noted; c) after T_{max}, if a measurable concentration appears after two consecutive BQLs, and after excluding measurement reasons, it should be replaced with BQL.

Blood drug concentration (C)-Time (T) data analysis: using PKCS, individual and mean C-T curves, and semi-logarithmic C-T curves are plotted separately; listing the mean drug concentration, standard deviation, quartiles, maximum value, minimum value, and coefficient of variation at each time point.

PK parameter analysis: PKPS will be used to calculate the PK parameters of each subject, such as AUC, C_{max}, CL, $T_{1/2}$, etc., using a non-compartmental model. At the same time, the arithmetic mean, standard deviation, coefficient of variation, median, maximum, minimum, and geometric mean of each parameter will be calculated.

Dose proportionality analysis: the relationship between AUC, C_{max}, and dose will be analyzed using a power function model, with the linear discrimination criterion being the 90% CI of the slope containing 1.

6. Immunogenicity analysis

The incidence and occurrence time of binding antibodies and neutralizing antibodies will be calculated, the antibody titers will be measured, and the proportion of positive antibodies will be determined.

XIV. Ethical considerations

1. Ethical norms

Prior to the implementation of this clinical study protocol, approval from the Ethics Committee is required. The sponsor and investigator must submit the protocol, informed consent forms, and other ethical review documents in accordance with ethical requirements to obtain the Ethics Committee's approval for conducting the clinical study. The approval from the Ethics Committee must be obtained before the clinical study can commence. During the clinical study, any issues related to the safety of the study, such as changes to the clinical study protocol or

informed consent forms for the subjects, as well as SAEs occurring in the study, must be approved or documented by the Ethics Committee.

2. Informed consent

The informed consent site is located at the Seventh Medical Center of the Chinese PLA General Hospital. Informed consent begins before the subject agrees to participate in the clinical study and continues throughout the entire process of the clinical study. The subject or their legal representative will be asked to review the informed consent form approved by the ethics committee and have 48 hours to fully consider it. The investigator or study physician informs the subject or their legal representative about the clinical study, the risks and potential benefits of the study, and answers any questions that the subject or their legal representative may have. The subject or their legal representative can only begin participating in the study after signing the informed consent form. During the entire clinical study, the subject may withdraw consent at any time. Even if they refuse to participate in this study, their rights and interests will be fully protected, and the quality of their medical care will not be affected in any way. A copy of the informed consent form must be provided to the subject or their legal representative.

XV. Confidentiality of information

Protection of subject privacy

During the course of this study, every effort will be made to protect the personal privacy of all subjects. Investigator-related documents, study reports, publications, and any other publicly disclosed materials must not include the names of subjects, unless required by law. The collection, transmission, processing, and storage of subject information will comply with relevant laws and regulations to ensure that subjects' personal information is not leaked.

In order to protect the privacy of the subjects, each subject will be assigned a unique, subject-specific code. Any subject records or datasets submitted to the sponsor must include this code and must not include the subject's name. The investigative site will maintain a list of subjects enrolled in this study, linking the subject's code to their true identity.

XVI. Subjects

Name	Title/profession	Task	GCP training (time)
Zhi-Chun Feng	Chief physician/clinical	Overall study design	January 25, 2021
Xiu-Wei Ma	Associate chief physician/clinical	Subject enrollment, study	Noveber 24, 2018
Yong-Xia Wang	Attending physician/clinical	Subject observation, follow-up	January 25, 2021
Yu Hou	Associate chief physician/clinical	Subject enrollment, study	Noveber 24, 2018
Jie Liu	Supervisor/nursing	Drug management, sample collection	September 16, 2017

17. References

1. NMPA. Guiding principles for non-clinical study and evaluation of gene therapy products (draft for soliciting opinions). February 2021.

2. NMPA. Technical guidance principles for long-term follow-up clinical studies of gene therapy products (draft for solicitation of comments). May 2021.

3. Beijing Medical Association Rare Disease Branch, Beijing Medical Association Medical Genetics Branch, et al. Expert consensus on multidisciplinary management of spinal muscular atrophy. Chin Med J. 2019;99:1460-6.

4. Beijing Medical Association Medical Genetics Branch, Beijing Rare Disease Diagnosis, Treatment, and Protection Association. Expert consensus on genetic diagnosis of spinal muscular atrophy. Chin Med J. 2020;100:3130-40.

5. Mendell JR, Al-Zaidy S, Shell R, Arnold WD, Rodino-Klapac LR, Prior TW, et al. Single-dose gene-replacement therapy for spinal muscular atrophy. N Engl J Med. 2017;377:1713-21.

6. CDER. Clinical Review(s) (application number: 213535orig1s000). 2020.

7. Sumner CJ, Paushkin S, Ko CP. Spinal muscular atrophy: disease mechanisms and therapy. 2016.

8. Kolb SJ, Kissel JT. Spinal muscular atrophy. Neurol Clin. 2015;33:831-46.

9. Study of intrathecal administration of onasemnogene abeparvovec-xioi for spinal muscular atrophy (STRONG)

https://clinicaltrials.gov/ct2/show/NCT03381729?term=Zolgensma&draw=2&rank=5.

Appendix 1: Definitions, measures, and follow-up requirements for liver safety and laboratory test trigger parameters

Individuals exposed to the drug without showing signs of liver injury (mainly determined by elevated GGT and transaminase levels) are referred to as "tolerators", while those who experience transient liver injury but adapt are called "adaptors". According to international clinical experience and toxicological studies of investigational drugs, elevated liver injury biomarkers such as transaminases are common adverse reactions to AAV drug delivery, usually mild and controllable. However, in some subjects, the elevation of these biomarkers may be a harbinger of more severe potential outcomes, and these individuals are unable to adapt, making them prone to progressive and severe liver injury, commonly referred to as drug-induced liver injury (DILI). This type of injury is closely related to drug dosage, delivery genes, and the carrier used. When transaminase levels exceed three times the baseline value at enrollment, more frequent monitoring should be implemented to determine whether the subject is an adaptor or a susceptible individual.

The laboratory test abnormality limits for potential DILI cases depend on the baseline values and underlying diseases of each subject. Investigators should further evaluate subjects with the following laboratory test abnormalities according to potential DILI cases (Hy's Law) to definitively determine the etiology of the abnormal laboratory values, as shown in the table below.

Table 1. Laboratory test result limits for liver events

	**Definitions and limits**
Liver-related laboratory test findings or events	3 × baseline < ALT/AST baseline ≤ 5 × baseline
	ALT or AST baseline > 5 × baseline
	ALP > 2 × baseline
	TBL > 2 × baseline
	Potential Hy's Law cases (defined as ALT or AST greater than 3 × baseline and TBL > 2 × baseline (mainly conjugated bilirubin), with ALP not significantly elevated to > 2 × baseline)
	Any clinical event of jaundice
	ALT or AST > 3 × baseline, accompanied by general malaise, fatigue, abdominal pain, nausea or vomiting, or rash with eosinophilia
	Any AE that may indicate hepatotoxicity[a]
[a]These events include: liver failure, liver fibrosis and cirrhosis, as well as other liver damage-related symptoms; non-infectious hepatitis, benign, malignant, and unspecified liver tumors	

Table 2. Follow-up requirements for liver events

Criteria	Actions to be taken	Follow-up monitoring
Potential Hy's Law Cases	Interrupt study treatment immediately	ALT, AST, TBL, Alb, PT/INR, ALP, γGT, until the issue is resolved (frequency to be determined at the investigator's discretion)
	Hospitalization	
	Assess for causality	
	Complete liver CRF	
AST or AST > 5 × baseline<br>> 3 × baseline and INR > 1.5<br>AST or AST > 3 × baseline, accompanied by symptoms	Interrupt study treatment immediately	ALT, AST, TBL, Alb, PT/INR, ALP, γGT, until the issue is resolved (frequency to be determined at the investigator's discretion)
	Hospitalization	
	Assess for causality	
	Complete liver CRF	
	Repeat liver function test (LFT) over 48 h	
Aspartate aminotransferase (AST) or AST > 3 × baseline to ≤ 5 × baseline, asymptomatic	Repeat LFT within 1 wk	The investigator, at their discretion, will conduct LFT monitoring within 1 to 4 wk
	If an increase in values is confirmed, perform close monitoring of the patient	

In suspected Hy's Law cases, in addition to repeated measurements of AST, ALT, and TBili, additional laboratory tests should include albumin, creatine kinase (CK), direct and indirect bilirubin, gamma-glutamyl transferase (GGT), prothrombin time (PT)/international normalized ratio (INR), total bile acids, and alkaline phosphatase. It is also advisable to collect a separate tube of coagulated blood and one of anticoagulated blood for further examination at the time of initial abnormality recognition, as needed for concurrent further analysis to determine the etiology. A detailed medical history should be obtained, including relevant information (acetaminophen, alcohol, recreational drugs and supplements, family history, travel history, contact with jaundiced patients, surgical history, transfusion history, history of liver disease or allergic diseases, and potential exposure to chemicals). Further investigations may be required for hepatitis A, B, C, D, and E infections and liver imaging (such as bile duct).

All cases with confirmed elevated AST/ALT and TBII levels according to the aforementioned laboratory criteria, should be considered as potential DILI (Hy's Law) cases if no other cause for the LFT abnormalities has been identified. These potential cases will be reported as SAEs, regardless of whether all test results to determine the cause of LFT abnormalities are available. Only when all reasonably conducted test results have been obtained and other causes have been ruled out, the potential DILI (Hy's Law) cases will be considered as confirmed cases.

Appendix 3. Schedule of assessment

Period	Screening baseline	Dosing period					Observation period		Follow-up period						Long-term follow-up
	Within 7 d	Week 1	Week 1	Week 1	Week 1	Week 1	Week 2	Week 4	Week 6	Week 8	Week 12	Week 16	Week 28	Week 52	5 y
		0 h, D0	4 h, D0	D1	D3	D6	D14	D28	D42	D56	D84	D112	D196	D364	
**Number of follow-up visits (V)**	**V1**	**V2**					**V3**	**V4**	**V5**	**V6**	**V7**	**V8**	**V9**	**V10**	
**Window period**			**± 5 min**	**± 1 h**	**± 2 h**	**± 12 h**	**± 3 d**	**± 3 d**	**± 6 d**	**± 6 d**	**± 9 d**	**± 10 d**	**± 15 d**	**± 30 d**	
Informed consent	X														
Medical history/demography	X														
Vital signs	X		X	X	X	X	X	X	X	X	X	X	X	X	X
Physical examination	X					X	X	X	X	X	X	X	X	X	X
Hematology	X					X	X	X	X	X	X	X	X	X	X
Blood chemistry	X					X	X	X	X	X	X	X	X	X	X
Urinalysis	X					X	X	X	X	X	X	X	X	X	X
Coagulation	X					X	X	X	X	X	X	X	X	X	
ECG	X					X	X	X	X	X	X	X	X	X	
Echocardiography	X					X		X			X			X	
**Period**	**Screening**	**Dosing period**					**Observation**		**Follow-up period**						**Long-term**

	**Baseline**						**Period**								**Follow-up**
	Within 7 d	Week 1	Week 1	Week 1	Week 1	Week 1	Week 2	Week 4	Week 6	Week 8	Week 12	Week 16	Week 28	Week 52	5 y
		0 h, D0	4 h, D0	D1	D3	D6	D14	D28	D42	D56	D84	D112	D196	D364	
Chest x-ray	X														
Infectious disease examination	X														
Pulmonary function assessment	X													X	
Eligibility assessment	X														
Enrollment	X														
Dosing		X													
Prednisolone[a]	X	X													
Immunogenicity[b]		1 h before dosing					X			X		X			X
PK (viral load) blood sampling		1 h before dosing				X	X	X	X	X	X	X		X	X
Electromyography (MUNE)	X					X		X		X		X	X	X	
MRI (muscle)	X													X	

**Period**	**Screening baseline**	**Dosing period**					**Observation period**		**Follow-up period**						**Long-term follow-up**

	Within 7 d	Week 1	Week 1	Week 1	Week 1	Week 1	Week 2	Week 4	Week 6	Week 8	Week 12	Week 16	Week 28	Week 52	5 y
		0 h, D0	4 h, D0	D1	D3	D6	D14	D28	D42	D56	D84	D112	D196	D364	
Neuromuscular function assessment (HFMSE)	X							X		X	X	X	X	X	X
Neuromuscular function assessment (RULM)	X							X		X	X	X	X	X	X
AEs	X	X	X	X	X	X	X	X	X	X	X	X	X	X	X
Concomitant medications	X														

[a]X[1] Prednisolone, dosing regimen: 1 mg/kg/day, < 60 mg/day; oral administration, given from Day -1 to Day 28, followed by a gradual dose reduction starting from Week 5 until discontinuation (dose reduction and discontinuation date determined by the investigator based on clinical practice); [b]immunogenicity involves: AAV9 neutralizing antibodies, AAV9 binding antibodies, SMN binding antibodies; for cytokines, IL-2, IL-4, IL-6, IL-10, IFN-γ, TNF-α will be determined in serum; [c]screening and baseline assessments are combined
